# Nidogen‐1 Mitigates Ischemia and Promotes Tissue Survival and Regeneration

**DOI:** 10.1002/advs.202002500

**Published:** 2020-12-21

**Authors:** Aline Zbinden, Shannon L. Layland, Max Urbanczyk, Daniel A. Carvajal Berrio, Julia Marzi, Monika Zauner, Anne Hammerschmidt, Eva M. Brauchle, Katrin Sudrow, Simon Fink, Markus Templin, Simone Liebscher, Gerd Klein, Arjun Deb, Garry P. Duffy, Gay M. Crooks, Johannes A. Eble, Hanna K. A. Mikkola, Ali Nsair, Martina Seifert, Katja Schenke‐Layland

**Affiliations:** ^1^ Department of Bioengineering Eberhard Karls University Tübingen Tübingen 72076 Germany; ^2^ Department of Women's Health Research Institute for Women's Health Eberhard Karls University Tübingen Tübingen 72076 Germany; ^3^ Cluster of Excellence iFIT (EXC 2180) “Image‐Guided and Functionally Instructed Tumor Therapies” Eberhard Karls University Tübingen Tübingen 72076 Germany; ^4^ NMI Natural and Medical Sciences Institute at the University of Tübingen Reutlingen 72770 Germany; ^5^ Institute of Medical Immunology Charité Universitätsmedizin Berlin corporate member of Freie Universität Berlin Humboldt‐Universität zu Berlin Berlin 10117 Germany; ^6^ Charité Universitätsmedizin Berlin Institute of Health (BIH) Center for Regenerative Therapies Berlin (BCRT) Berlin 10178 Germany; ^7^ Center for Medical Research Department of Medicine II Eberhard Karls University Tübingen Tübingen 72076 Germany; ^8^ Department of Molecular Cell and Developmental Biology UCLA Los Angeles CA 90095 USA; ^9^ Eli and Edythe Broad Stem Cell Research Center UCLA Los Angeles CA 90095 USA; ^10^ Molecular Biology Institute UCLA Los Angeles CA 90095 USA; ^11^ Jonsson Comprehensive Cancer Center UCLA Los Angeles CA 90024 USA; ^12^ Department of Medicine/Cardiology Cardiovascular Research Laboratories David Geffen School of Medicine at UCLA Los Angeles CA 90095 USA; ^13^ Anatomy and Regenerative Medicine Institute School of Medicine College of Medicine Nursing and Health Sciences National University of Ireland Galway Galway H91TK33 Ireland; ^14^ Department of Pathology and Laboratory Medicine, and Pediatrics David Geffen School of Medicine at UCLA UCLA Los Angeles CA 90095 USA; ^15^ Institute of Physiological Chemistry and Pathobiochemistry University of Münster Münster 48149 Germany; ^16^ DZHK (German Centre for Cardiovascular Research) partner site Berlin Berlin 10117 Germany

**Keywords:** diabetes, ischemia, myocardial infarction, nidogen‐1, pancreatic *β*‐cells

## Abstract

Ischemia impacts multiple organ systems and is the major cause of morbidity and mortality in the developed world. Ischemia disrupts tissue homeostasis, driving cell death, and damages tissue structure integrity. Strategies to heal organs, like the infarcted heart, or to replace cells, as done in pancreatic islet *β*‐cell transplantations, are often hindered by ischemic conditions. Here, it is discovered that the basement membrane glycoprotein nidogen‐1 attenuates the apoptotic effect of hypoxia in cardiomyocytes and pancreatic *β*‐cells via the *α*v*β*3 integrin and beneficially modulates immune responses in vitro. It is shown that nidogen‐1 significantly increases heart function and angiogenesis, while reducing fibrosis, in a mouse postmyocardial infarction model. These results demonstrate the protective and regenerative potential of nidogen‐1 in ischemic conditions.

## Graphical abstract

Nidogen‐1 functionalized gels increase cell and tissue survival and function in ischemic conditions via a synergic combination of improved angiogenesis, cell protection, immunomodulation, and reduced fibrosis. A preclinical mouse model of myocardial infarction shows an increase in heart function and demonstrates the clinical potential of a Nidogen‐1 as an off‐the‐shelf therapy.

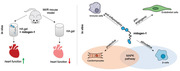

## 1 Introduction

Ischemic injury due to the disruption of blood flow can lead to irreversible tissue injury precipitating to neurologic stroke, limb ischemia or myocardial infarction (MI).^[^ ^1^ ^]^ Ischemic conditions also disrupt regenerative and protective therapies to attenuate cell death and restore organ function, particularly in applications where cell engraftment is required. Strategies to protect cells, modulate the immune response, and repair the tissue milieu within ischemic environments are therefore of critical importance.

The current clinical and preclinical stage strategies that seek to restore organ function post‐ischemia include the injection of cells, growth factors, small molecules, hydrogels or decellularized extracellular matrix (ECM) proteins.^[^ ^2^, ^3^, ^4^ ^]^ The ECM is the 3D noncellular component of tissues and organs. It consists of mostly water, proteins, and polysaccharides, and provides the biophysical scaffolding for tissues. The ECM also offers important biochemical and mechanical cues influencing cell homeostasis and differentiation.^[^ ^5^ ^]^ Ischemia leads to pathological ECM remodeling, resulting in fibrosis due to the deposition of fibrillar proteins, such as collagens type I (COL1) and III, leading to fibrotic scarring.^[^ ^6^ ^]^ The alteration of ECM proteins can lead to cellular mutations, transdifferentiation or apoptotic death.^[^ ^7^ ^]^ Basement membranes (BMs) are crucial ECM structures created by networks of collagen type IV (COL4) and laminins (LAM), which are linked by nidogen‐1 (NID1). Other macromolecules integrate into BMs to give them unique functions in different tissues.^[^ ^8^ ^]^ BMs are essential for tissue development and homeostasis.^[^ ^9^ ^]^ In previous work, we demonstrated the cardiogenic effect of COL4 and LAM in vitro,^[^ ^10^ ^]^ as well as the role of NID1 in human embryonic stem cell (hESC) assembly.^[^ ^11^ ^]^ Therefore, we asked whether a single BM protein could have a functional impact on tissue protection or regeneration.^[^ ^12^ ^]^

To address the condition of MI, we analyzed the expression of BM proteins during heart development to identify candidates that could protect cardiac tissues during ischemia and support regeneration. NID1, also known as entactin‐1,^[^ ^13^ ^]^ was selected as the lead candidate as it was the most prominent BM protein in hESC‐derived cells that were differentiated to the cardiovascular lineages. In vitro and in vivo studies uncovered a novel protective function of NID1 in the cardiovascular system. We also documented a beneficial effect of NID1 on immune cells, which is an essential component of the regenerative process for therapeutic approaches.

To test if NID1 may have a similar effect on other organ systems, therefore potentially supporting a regenerative therapy that is otherwise hindered by ischemic conditions, we choose a model of pancreatic beta cell transplantation, which is a therapy for type 1 diabetes where up to 60% of beta‐cell‐containing islets fail to engraft due to ischemia at the transplant location and the adverse reaction of the immune system.^[^ ^14^ ^]^ Here, we demonstrated the protective and functional effect of NID1 on pancreatic beta cells in an ischemia model. Our studies uncovered a novel protective function of NID1 in multiple organ systems in vitro and in vivo and elucidate potential integrin‐driven mechanisms of action.

## 2 Results

### 2.1 NID1 Is Identified as a Candidate for Cardiovascular Regenerative Approaches

**Figure 1:**
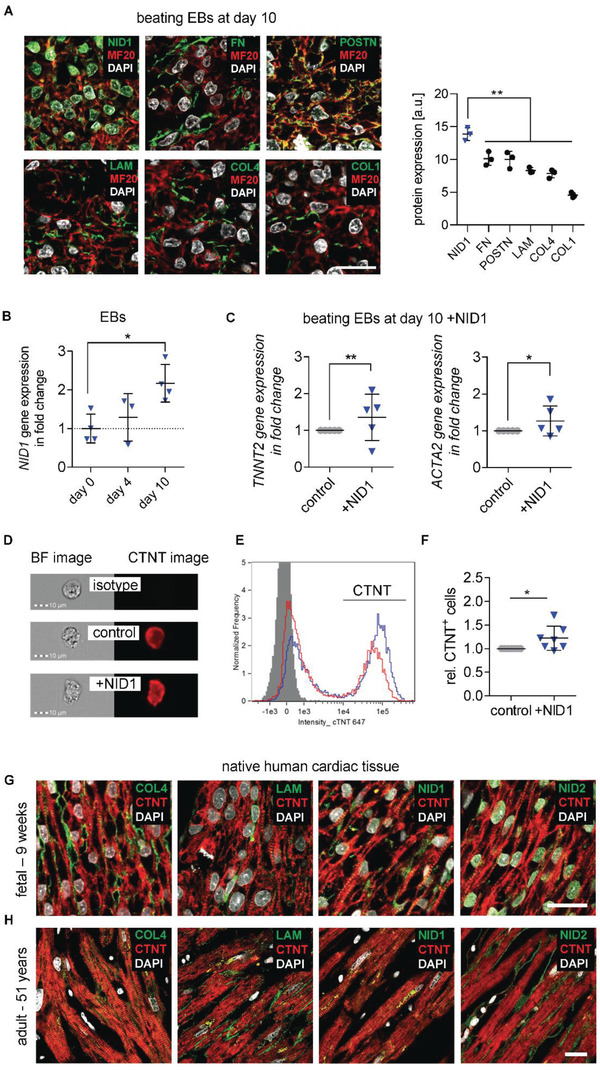
ECM BM protein NID1 is identified as a candidate for regenerative approaches. A) IF staining and semiquantification of BM proteins NID1, FN, POSTN, LAM, COL4, and COL1, as well as DAPI and MF20 in day‐10 beating EBs. Gray value intensities (GVI) of IF images were normalized to the laser intensity (*n* = 3), one‐way ANOVA with Tukey's multiple comparisons test. B) qPCR analysis of *NID1* expression on day 0 (undifferentiated hESCs), and after 4 and 10 days (beating EBs) of cardiovascular differentiation. Data are normalized to the average of day 0 and shown as fold change (*n* = 3–4); one‐way ANOVA with Tukey's multiple comparisons test. C) qPCR analysis of *TNNT2* and *ACTA2* gene expression within EBs at day 10 of cardiovascular differentiation without (control) and with NID1 (*n* = 5); Kolmogorov–Smirnov *t*‐test. D) Bright field (BF) and CTNT IF images of single cells derived from EBs that were cultured for 10 days without (control) and with NID1 acquired with 40× magnification using imaging flow cytometry. An isotype control is provided. E) Representative histogram of cells that were cultured for 10 days with (blue) or without (red) NID1. The isotype control is shown in gray. F) Quantification of the data obtained by imaging flow cytometric analysis showing the relative amount of CTNT^+^ cells derived from EBs that were cultured for 10 days without (control) and with NID1 (*n* = 7); Kolmogorov–Smirnov *t*‐test. G,H) IF staining of BM proteins COL4, LAM, NID1, NID2, as well as DAPI and sarcomeric myosin CTNT within human (G) fetal heart sections (9 weeks postgestation) and (H) adult heart tissue (51 years). Scale bars: 20 µm. *p < 0.05, **p < 0.01, ***p < 0.001, and ****p < 0.0001.

To identify potential therapeutic cardiovascular candidates, the expression of ECM BM proteins was investigated in differentiating hESC‐derived embryoid bodies (EBs). We used a modified cardiovascular differentiation protocol for the formation of EBs from the H9 hESC line (Figure S1, Supporting Information).^[^ ^15^, ^16^ ^]^ Semiquantification of ECM immunofluorescence (IF) staining within spontaneously beating day‐10 EBs showed a significantly higher expression of NID1 compared with other well‐investigated ECM proteins such as fibronectin (FN), periostin (POSTN), LAM, COL4, and COL1 (**Figure** **1**A).^[^ ^10^, ^11^, ^12^, ^13^, ^14^, ^15^, ^16^, ^17^, ^18^ ^]^ In addition, NID1 gene expression significantly increased during cardiovascular differentiation (Figure 1B).

To assess if NID1 has a beneficial effect during cardiovascular differentiation, we produced recombinant full length human NID1, available upon request, (Figure S2, Supporting Information) and supplemented it to hESCs that were differentiating toward the cardiovascular lineages. The supplementation of NID1 increased gene expression for cardiac (cardiac troponin T (*TNNT2)*) and smooth muscle (smooth muscle *α*2‐actin (*ACTA2*)) cell markers (Figure 1C). Image‐Stream analyses revealed the presence of a significantly higher number of cardiac muscle troponin T (CTNT)^+^ cells within the NID1‐treated cultures when compared with the controls, indicating a potential cardioinductive or cardioprotective effect of NID1 (Figure 1D–F).

The presence of NID1 in native human cardiac tissue was verified both during development (9‐, 12‐, 17‐week embryonic) and in the adult (18 and 51 years) by IF staining (Figure 1G,F; Figure S3A, Supporting Information). The positive correlation of NID1 with cardiovascular development and its presence in human cardiac tissue nominated NID1 as a potential candidate for regenerative and reparative therapies.

### 2.2 NID1 Improves Heart Function Post Myocardial Infarction

Regenerative and remodeling approaches for post‐MI therapies, to modulate infarction size and scar formation, aim to protect cardiovascular cells from the ischemic environment. We thus asked whether the potential remodeling properties of NID1 could improve the outcome of an MI and reperfusion (MI/R) mouse model. Ischemia/reperfusion was conducted through the ligation of the left anterior descending (LAD) artery in C57BL/6J mice as previously described.^[^ ^19^ ^]^ Directly after reperfusion, three groups of mice received a single treatment of five injections in the infarct border zone of either saline as a procedure control, the hyaluronic acid (HA) gel carrier as a carrier control, or NID1 within the HA carrier gel (NID1 + HA).

**Figure 2:**
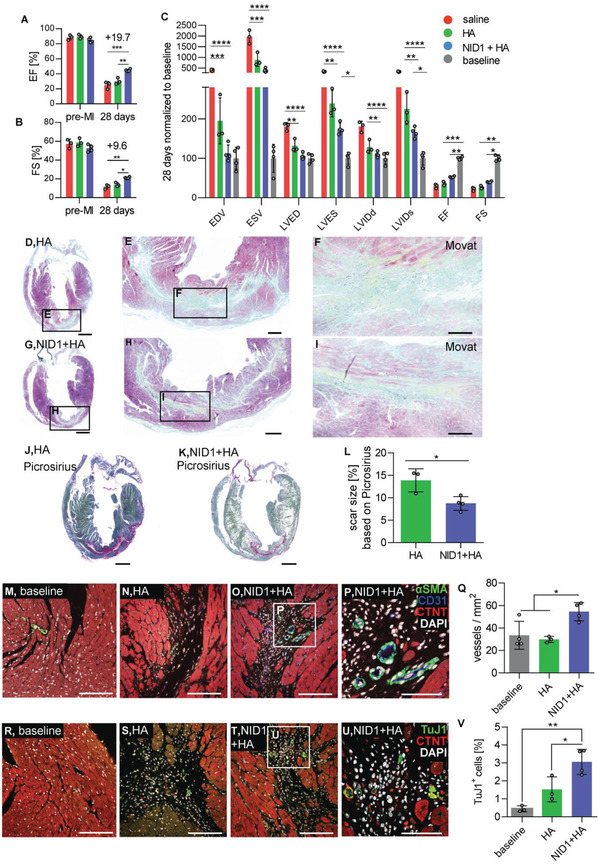
NID1 increases heart function post‐MI. A–C) Echocardiography analysis: absolute values of (A) EF and (B) FS after intracardiac injections of saline, HA and 50 µg mL^−1^ NID1 + HA at 28 days post‐MI/R, and C) parameters were normalized to the baseline at 28 days post‐MI/R. Echocardiography data were analyzed by one‐way ANOVA with Tukey's multiple comparisons test. D–I) Movat pentachrome staining of representative sections of (D–F) HA‐ and (G–I) NID1 + HA‐treated hearts after 28 days post‐MI/R with scar tissue stained in green and yellow. Scale bars: (D,G) 1 mm, (E,H) 200 µm, and (F,I) 100 µm. J,K) Picrosirius Red and Fast Green staining of representative (J) HA‐ and (K) NID1 + HA‐treated heart sections with scar tissue stained in pink. Scale bars: 1 mm. L) Quantification of scar size in Picrosirius Red‐ and Fast Green‐stained serial sections. Whole‐heart scans of every tenth slide throughout the whole heart were analyzed. M–P) Confocal images of *α*SMA, CD31, and CTNT IF staining of representative (M) baseline, (N) HA‐, and (O) NID1 + HA‐treated heart sections obtained with a 25× magnification (scale bar: 100 µm), and with a P) 63× magnification (scale bar; 50 µm). Q) Quantification of vessel density within the infarct area using images obtained with a 25× magnification. R–U) Images of TuJ1 and CTNT IF staining of representative (R) baseline, (S) HA‐, and (T) NID1 + HA‐treated tissue sections obtained with a 25× magnification (scale bar equal 100 µm), and a (U) 63× magnification (scale bar: 50 µm). V) Quantification of TuJ1^+^ cells within the infarct area. For all MI/R studies saline mice (*n* = 3), HA mice (*n* = 3), NID1 + HA‐treated mice (*n* = 4) were used, unpaired *t*‐test. *p < 0.05, **p < 0.01, ***p < 0.001, ****p < 0.0001. LVIDd: left ventricular internal dimension at end diastole, LVIDs: left ventricular internal dimension at end‐systole, LVED: left ventricle end‐diastolic diameter, LVES: left ventricle end‐systolic diameter, EDV: end‐diastolic volume, ESV: end‐systolic volume, EF: ejection fraction, FS: fractional shortening.

Echocardiography 28 days post MI/R revealed a significant increase of heart ejection fraction (EF) by 19.7% (NID1 + HA 44.6% ± 1.6% versus saline 25% ± 4.8%, *p* < 0.001) (**Figure** **2**A) and fractional shortening (FS) by 9.6% (NID1 + HA 21.1% ± 0.8% versus saline 11.5% ± 2.3%, *p* < 0.01) (Figure 2B). Left ventricular (LV) volume and diameter (end‐systolic volume (ESV), left ventricle end‐systolic diameter (LVES), and left ventricular internal dimension at end ‐systole (LVIDs)) were all significantly improved in the NID1 + HA‐treated hearts when compared with the controls (*p* < 0.0001) (Figure 2C). Significant differences between NID1 + HA and HA treatments were found in EF and FS, as well as in LV volume and diameter. Strikingly, NID1 + HA treatment resulted in a complete recovery in end‐diastolic volume (EDV), left ventricular end‐diastolic diameter (LVED), and left ventricular internal dimension at end ‐diastole (LVIDd) when compared with the pre‐MI/R baseline (Table S1, Supporting Information); however, the HA‐treated hearts also showed a recovery in these values, which should be taken into consideration as the HA carrier gel may have contributed to the positive effect in these parameters.

Mouse hearts from the HA and NID1 + HA‐treated groups were excised after the final echocardiography and processed. Russel–Movat pentachrome staining identified qualitative differences between the ECM‐rich infarct zone and the surrounding tissue with intact cardiac muscle (Figure 2D–I). Picrosirius Red and Fast Green staining of serial sections throughout the whole heart (Figure 2J,K) were analyzed to identify and quantify scar tissue formation based on hue, saturation, and value (HSV) histograms (Figure S3B, Supporting Information). Scar tissue constituted 13.9% ± 2.6% of the HA‐treated hearts and 8.8% ± 1.5% of the NID1 + HA‐treated hearts, which is a 37% absolute reduction of scar tissue in the entire heart, not only the LV (Figure 2L).

IF staining of α‐smooth muscle actin (*α*SMA) and CD31 was performed to study the effect of the NID1 + HA treatment on angiogenesis within the infarct zone (Figure 2M–P). The density of *α*SMA^+^/CD31^+^ vessels in the scar area was significantly increased in the NID1 + HA treatment group when directly compared with the baseline and HA‐treated tissues (Figure 2Q).

It has been recently demonstrated that reinnervation is critical for mammalian cardiac regeneration.^[^ ^20^ ^]^ We used the neuronal marker β‐tubulin 3 (TuJ1) to investigate the potential of NID1 + HA to increase nerve protection and innervation of infarcted tissue (Figure 2R–U). Interestingly, we identified a significant twofold increase of TuJ1^+^ cells in the infarct area of the NID1 + HA‐treated hearts compared with the baseline and HA‐treated hearts (Figure 2V).

To investigate the scar tissue quality in the infarct areas, Raman microspectroscopy and Raman imaging were employed as noninvasive marker‐free techniques to differentiate biochemical spectral fingerprints as previously demonstrated by our group.^[^ ^21^ ^]^ True component analysis (TCA) and principal component analysis (PCA) identified molecular differences and spatial distribution of spectral information corresponding to myocardium, DNA, and scar tissue (Figure S4A,B, Supporting Information). Interestingly, PCA analysis of the scar fingerprint of the NID1 + HA‐treated mice showed peaks associated with glycogen (497 cm^−1^), porphyrin (1513, 1557, and 1612 cm^−1^), and DNA (1093 cm^−1^), while the scar fingerprint of the control mice was dominated by peaks assigned to collagens (858, 940, and 1248 cm^−1^), which indicates that the scar tissue in the treated mice resembled the molecular fingerprint of the noninfarcted myocardium connective tissue (Extended Data in Figure S4C–G and Table S2, Supporting Information). Taken together, these data demonstrate that NID1 positively affects heart function, angiogenesis, scar size, and scar tissue quality in a therapeutic model.

### 2.3 NID1 Protects Cardiovascular Cells in an Ischemia In Vitro Model

**Figure 3:**
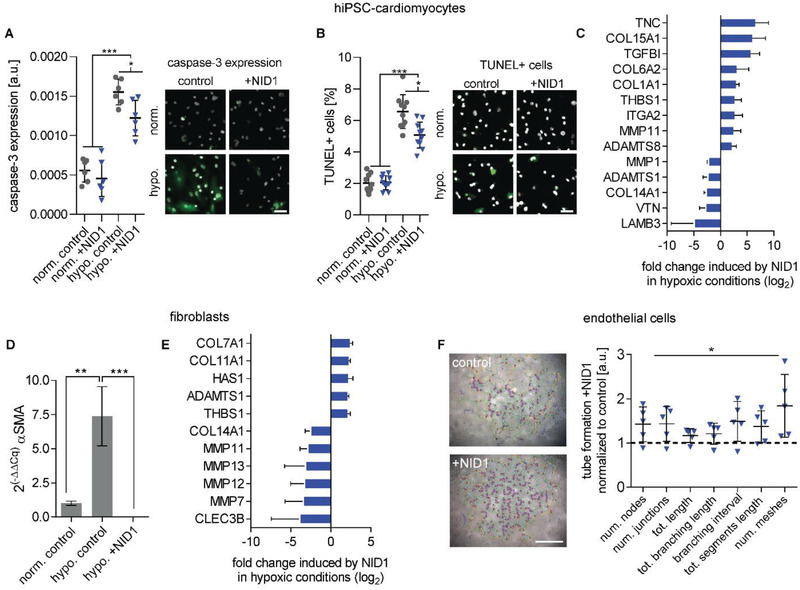
NID1 mitigates the effects of hypoxia on cardiovascular cells. A,B) Protective effect of NID1 on hiPSC‐CMs shown by (A) cleaved caspase‐3 staining and semiquantification (*n* = 6) and (B) by number of TUNEL^+^ cells over total cell number (*n* = 10); one‐way ANOVA with Tukey's multiple comparisons test. C) qPCR array analysis of NID1‐treated hiPSC‐CMs under hypoxic conditions with a focus on matrix production, degradation, and regulation (*n* = 3). D) qPCR analysis for the expression of *α*SMA in control fibroblasts under normoxic and hypoxic conditions, as well as for NID1‐treated fibroblasts under hypoxic conditions (*n* = 3). One‐way ANOVA with Tukey's multiple comparisons test. E) qPCR array analysis of NID1‐treated fibroblasts under hypoxic conditions (identical qPCR array as in (C)) (*n* = 3). F) Tube formation assay performed using 1.5 × 10^4^ HUVECs/0.32 cm^2^ with serum‐reduced Matrigel without (control) or with NID1 (*n* = 4–5). Quantification of different tube formation parameters using the angiogenesis analyzer of the ImageJ software. Data are normalized to the Matrigel control (set as 1), one‐way ANOVA with Tukey's multiple comparisons test. For qPCR, gene regulation by NID1 is shown when fold‐regulation > |2|, *p*‐value < 0.05. *p < 0.05; **p < 0.01, ***p < 0.001, and ****p < 0.0001. Scale bars: 50 µm.

To elucidate the therapeutic effect of NID1 in vitro, human induced‐pluripotent stem cell‐derived (hiPSC) iCell cardiomyocytes (CMs) were investigated in a hypoxia (1% oxygen) ischemia‐like in vitro model. For two days, NID1‐treated and control hiPSC‐CMs were cultured under normoxic and hypoxic conditions and evaluated for cell death via the expression of cleaved caspase‐3 and the number of TUNEL^+^ cells (**Figure** **3**A,B). NID1‐treated hiPSC‐CMs had a significantly lower expression of cleaved caspase‐3 and a reduced the number of TUNEL^+^ cells in hypoxic conditions when compared with nontreated controls. No changes were observed in normoxic cultures in the presence of NID1.

PCR array analysis of the expression of human ECM and adhesion molecules in NID1‐treated hiPSC‐CMs identified several significantly up and downregulated genes (Figure 3C). In hypoxic conditions, NID1‐treated hiPSC‐CMs showed significantly upregulated expression levels for *TNC, THBS1, ITGA2, ADAMTS8, MMP11*, and several collagens such as *COL1A1*, *COL6A2*, and *COL15A1*. Interestingly, *TGFB1* was significantly upregulated in both normoxic and hypoxic NID1‐treated hiPSC‐CMs (Figure S5A, Supporting Information). *LAMB3*, *VTN*, *COL14A1*, *ADAMTS1*, and *MMP1* were significantly downregulated in NID1‐treated hiPSC‐CMs under hypoxic conditions. Of the transcriptional changes in ECM‐associated genes in hiPSC‐CMs induced by hypoxic conditions, 28 genes out of a total of 89 genes tested were significantly differentially expressed compared to the normoxic groups. In NID1‐treated hiPSC‐CMs, transcriptional changes induced by hypoxia were minimal with only one differentially expressed gene (*COL14A1*) (Figure S5B, Supporting Information).

Hypoxia is known to induce a phenotypic switch from fibroblasts to myofibroblasts, which is the cell population producing the majority of the structural ECM proteins during fibrosis.^[^ ^22^ ^]^ The phenotypic switch to myofibroblasts can be assessed by *α*‐SMA expression and was observed in our control fibroblasts (Figure 3D). Interestingly, the expression of *α*‐SMA was significantly reduced in NID1‐treated fibroblasts. Gene expression analysis of fibroblasts showed that *MMP7*, *MMP11*, *MMP12*, *MMP13, COL14A1*, and *CLEC3B* were significantly downregulated by NID1; and *COL7A1*, *COL11A1*, *HAS1*, *ADAMTS1*, and *THBS1* were significantly upregulated by NID1 (Figure 3E). Exposure to hypoxic conditions resulted in significant up‐ or downregulation of 26 genes in the control group, while only 11 genes were impacted in NID1‐treated fibroblasts (Figure S5C, Supporting Information).

The MI/R model data showed an increased number of *α*SMA^+^/CD31^+^ vessels in the NID1‐treated infarcted hearts. To test the angiogenic potential of NID1, a human umbilical vein endothelial cell (HUVEC) tube formation assay was performed. NID1 significantly increased a variety of angiogenesis parameters including the total segment length, number of meshes, and total mesh area, as well as number of segments (Figure 3F).

Our data shows that NID1 supports angiogenesis and cardiovascular cell homeostasis during hypoxia and provides an antifibrotic effect on fibroblasts, elucidating the cellular events underlying the positive functional effect of NID1 in the therapeutic model.

### 2.4 NID1 Protects Pancreatic *β*‐Cells and Increases Insulin Secretion in an In Vitro Ischemia Model

**Figure 4:**
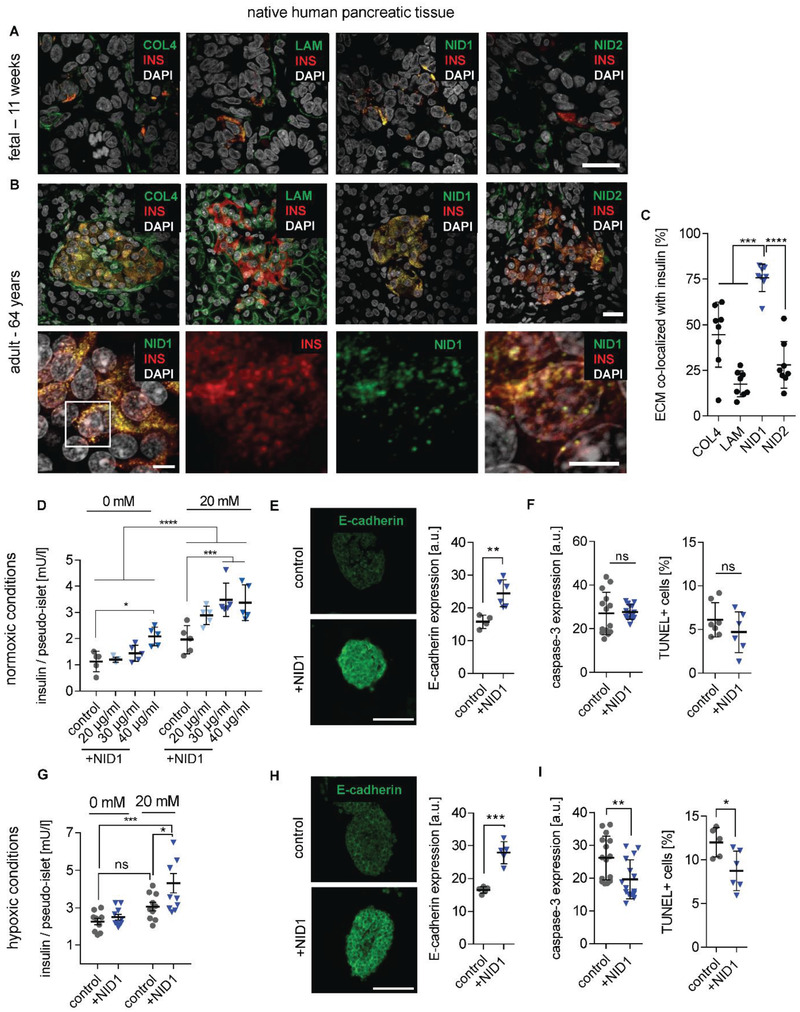
NID1 mitigates the effects of hypoxia on pancreatic *β*‐cells and increases insulin secretion. A,B) Expression pattern of BM proteins, insulin (INS), and DAPI in human (A) fetal (11 weeks postgestation) and (B) adult pancreas (64 years). Scale bars: 20 µm. Highly magnified images show the colocalization of NID1 with INS in native adult pancreas. Scale bar: 5 µm. C) Quantification of the colocalization of ECM proteins with INS (*n* = 10), one‐way ANOVA with Tukey's multiple comparisons test. D) GSIS response (with 0 × 10^−3^ and 20 × 10^−3^ m glucose) under normoxic conditions of human NID1‐treated pseudoislets in suspension at different concentrations: 20, 30, 40 µg mL^−1^ when compared with the control (PBS) (*n* = 5); two‐way ANOVA with Tukey's multiple comparisons test. E) E‐cadherin expression under normoxic conditions (*n* ≥ 5). F) Cell death under normoxic conditions via the detection of cleaved caspase‐3 (*n* = 14) and TUNEL^+^ cells (*n* = 7). G) GSIS response (with 0 × 10^−3^ and 20 × 10^−3^ m glucose) under hypoxic conditions of NID1‐treated pseudoislets at 30 µg mL^−1^ and normalized by live cells (*n* = 10); two‐way ANOVA with Tukey's multiple comparisons test. H) E‐cadherin expression under hypoxic conditions (*n* ≥ 5 m; unpaired *t*‐test. I) Protective effect of NID1 assessed by cleaved caspase‐3 expression (*n* ≥ 7). and via detection of TUNEL^+^ cells (*n* ≥ 4); unpaired *t*‐test. *p < 0.05; **p < 0.01, ***p < 0.001, and ****p < 0.0001.

We next asked if the protective and antifibrotic effect of NID1 during ischemia is organ‐specific or would be of benefit in other noncardiac therapeutic areas, such as islet transplantation, where ischemia and the survival of transplanted islets are a major therapeutic roadblock. The presence of BM proteins COL4, LAM, NID1, and nidogen‐2 (NID2) was confirmed in human 11‐week fetal and 64‐year adult pancreatic tissues by IF staining (**Figure** **4**A,B). Interestingly, in adult tissues, NID1 was spatially confined to insulin‐producing *β*‐cells in contrast to other BM proteins, suggesting that NID1 has a specific function in *β*‐cells (Figure 4C).

To determine if NID1 impacts *β*‐cell functionality, we established an in vitro hypoxia model using *β*‐cell aggregates, so‐called pseudoislets, using the conditionally immortalized human EndoC‐*β*H3 cell line (Figure S6A–I, Supporting Information). The impact of dosages of 20, 30, and 40 µg mL^−1^ NID1 on pseudoislet function was first assessed under normoxic conditions. NID1‐treated pseudoislets significantly increased insulin secretion at all dosages; with 30 µg mL^−1^ reaching the maximum effect, which was the dosage chosen for all further pseudoislet experiments (Figure 4D). IF staining of NID1‐treated pseudoislets cultured under normoxic conditions showed a significant increase in E‐cadherin when compared with the controls (Figure 4E). NID1 had no effect on cell death under normoxic conditions, assessed by the quantification of TUNEL^+^ cells and cleaved caspase‐3 staining (Figure 4F). In hypoxic conditions, NID1 rescued the loss of insulin secretion that was lost in control cultures, preserved the significant increase in E‐cadherin seen in normoxia, and significantly reduced cell death (Figure 4G–I).

Raman imaging with TCA analysis and multivariate curve resolution confirmed that NID1 enhances *β*‐cell function in hypoxia, particularly in the pseudoislet core, by an increase in mitochondrial function, insulin, and insulin‐transporting lipid vesicles (Figure S6J–P, Table S2, Supporting Information).^[^ ^23^ ^]^ These data demonstrate the protective and stimulative effect of NID1 on pancreatic *β*‐cells.

### 2.5 NID1 Modulates Immune Cell Responses toward Regeneration

**Figure 5:**
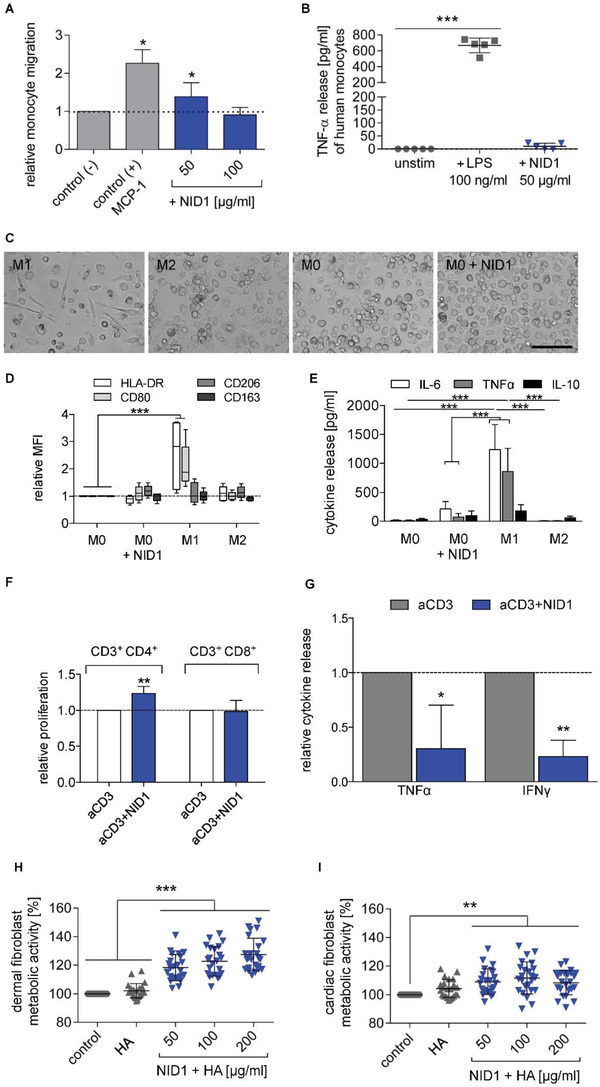
NID1 modulates immune cells. A) CD14^+^ monocyte migration in the presence of different NID1 concentrations (50, 100 µg mL^−1^) and the control protein MCP‐1 after 3 h. Cell migration is shown relative to the control (−) (*n* = 3 experiments with 9 single values); one‐way ANOVA with Dunnett's multiple comparison test. B) Potential endotoxin contamination was tested by adding 50 µg mL^−1^ NID1 or 100 ng mL^−1^ LPS in a monocyte culture for 24 h. Released TNF*α* was measured by ELISA (*n* = 5); Friedman test with Dunn's multiple comparison test. C) Representative images of M1‐ and M2‐type macrophages, unstimulated M0‐type macrophages and NID1‐treated M0‐type macrophages. Scale bar: 100 µm. D) Expression of polarization markers (HLA‐DR, CD80, CD206, CD163) was determined for M0‐type macrophages cultured for 24 h with 50 µg mL^−1^ NID1, M0, M1, and M2‐type macrophages. Mean fluorescence intensity (MFI) is shown relative to the untreated M0 macrophage control (set as 1) (*n* = 5); two‐way ANOVA with Dunnett's multiple comparison test. E) Release of IL‐6, TNF*α*, and IL‐10 after 24 h culture of M0 macrophages with NID1 (50 µg mL^−1^) and M0, M1 or M2 type cultures (*n* = 5); two‐way ANOVA with Tukey's multiple comparison post‐test. F) T cell proliferation after 5 days culture of human PBMCs with low‐dose aCD3 alone or combined with NID1 (50 µg mL^−1^) was tested in a CFSE‐based assay and measured by flow cytometry. Shown is the relative proliferation level of CD3^+^ CD4^+^ and CD3^+^ CD8^+^ T cells compared to the aCD3 control. G) Relative TNF*α* and IFN*γ* release of PBMC cultures after 5 days with either aCD3 alone or combined with NID1 (50 µg mL^−1^). Data for proliferation and cytokine release were analyzed by Kolmogorov–Smirnov *t*‐test, relative to the aCD3 control (*n* = 6). H) Cytotoxicity test using human dermal fibroblasts (EN ISO 10993) or I) cardiac fibroblasts. Cell viability was measured after treatment with control medium, HA hydrogel, and HA‐supplemented with NID1 (50, 100, and 200 µg mL^−1^) for 24 h. Cell viability is shown relative to the control (set as 100% viability) (*n* ≥ 22); one‐way ANOVA with Tukey's multiple comparisons test: *p < 0.05, **p < 0.01, ***p < 0.001, and ****p < 0.0001.

Our data nominated NID1 as a potential therapeutic candidate. Therefore, its interaction with immune cells of the innate and adaptive system was evaluated in vitro utilizing a previously described human‐based assay.^[^ ^24^ ^]^ Briefly, a chemotaxis assay was performed to measure the impact of NID1 on human CD14^+^ blood monocyte migration (**Figure** **5**A). CD14^+^ blood monocyte migration is an important process to clear the tissue of debris from apoptotic cells and disrupted ECM, thereby inducing normal tissue remodeling. Significant migration was detected for the 50 µg mL^−1^ NID1‐treated CD14^+^ monocytes compared with the negative control. Measuring the induced short‐term tumor necrosis factor alpha (TNF*α*) release from human monocytes showed negligible TNF*α* levels for a NID1 dosage up to 50 µg mL^−1^ compared with the lipopolysaccharide (LPS) control (Figure 5B).

To determine the influence of NID1 on macrophage polarization, unpolarized M0 macrophages were exposed to NID1 for 24 h. The macrophages were of rounded morphology with a tendency to arrange in clusters. This appearance was qualitatively more similar to an alternatively activated/regenerative (M2) macrophage, since classically activated/proinflammatory (M1) macrophages have a more elongated cell shape (Figure 5C). Flow cytometry studies detecting the surface molecules HLA‐DR, CD80, CD206, and CD163 confirmed that NID1‐treated macrophages did not develop an M1 phenotype (Figure 5D). Cytokine analysis of supernatants from NID1‐treated macrophages confirmed the absence of M1 macrophage induction. Rather, a profile was detected that is characteristic for M0 or M2 macrophages with low levels of IL‐6 and TNF*α* (Figure 5E).

T cell subsets with diverse functions can also influence the inflammatory milieu and the remodeling process, for example, post‐MI in heart tissue.^[^ ^25^ ^]^ Therefore, we studied the impact of NID1 on preactivated T cells by measuring its effect on anti‐CD3 (aCD3)‐induced proliferation of classical T cell subsets. NID1 provided an additional trigger to CD4^+^ T cells, but not to the cytotoxic CD8^+^ T cell fraction (Figure 5F). The release of the proinflammatory cytokines TNF*α* and IFN*γ* was significantly reduced in 5‐day cultures of NID1‐treated aCD3‐activated immune cells (Figure 5G). A standardized cytotoxicity test was performed according to EN ISO 10993. With this test, we assessed the metabolic activity of primary‐isolated human dermal fibroblasts to determine a potential cytotoxic effect of NID1 as requested by certification bodies such as the European Medicines Agency (Figure 5H). As a complementary test, cytotoxicity of NID1 was also investigated using human primary‐isolated cardiac fibroblasts (Figure 5I). We observed no cytotoxic effect due to NID1 exposure in the tested concentrations of 50, 100, and 200 µg mL^−1^. Interestingly, we noted an increased metabolic activity of both dermal and cardiac fibroblasts in the NID1‐treated groups compared with controls. These data show the regenerative immunomodulatory effect of NID1 in a human in vitro system and the safety of NID1 at dosages up to 50 µg mL^−1^, supporting the therapeutic potential of NID1.

### 2.6 NID1 Signals via Integrin *α*v*β*3 and Activates the MAPK Pathway

**Figure 6:**
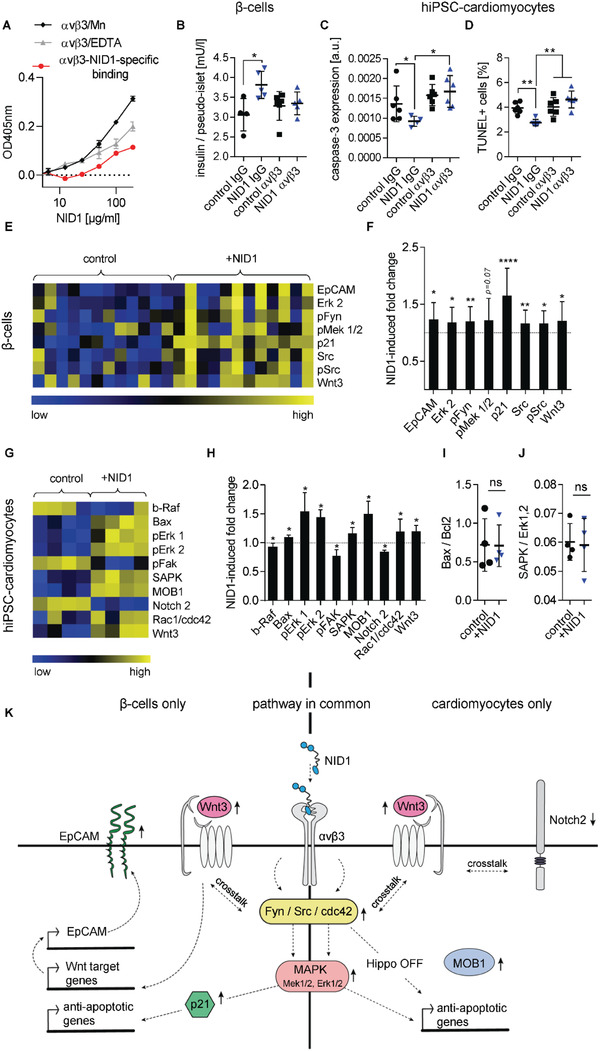
NID1 signals via *α*v*β*3 and activates the MAPK pathway in *β*‐cells and cardiomyocytes. A) Divalent cation‐dependent and dose‐dependent binding of *α*v*β*3 with NID1. B) Blocking of *α*v*β*3 in NID1‐treated and control pseudoislets under normoxic conditions. Functionality assessment by glucose stimulation at 20 × 10^−3^ m glucose (*n* ≥ 4); one‐way ANOVA with Tukey's multiple comparisons test. c,d) Blocking of *α*v*β*3 in NID1‐treated and control hiPSC‐CMs by a *α*v*β*3 antibody under hypoxic conditions. Protective effect of NID1 assessed by C) cleaved caspase‐3 (*n* ≥ 4) and D) TUNEL assay (*n* = 6); one‐way ANOVA with Tukey's multiple comparisons test. E–H) DigiWest‐based protein expression analysis of significantly regulated proteins in NID1‐treated E,F) pseudoislets (*n* = 12) and G,H) hiPSC‐CMs (*n* = 4) under hypoxic conditions compared with their respective controls. Data are (E,G) shown as column‐wise and color‐coded heatmap from the lowest (blue) to the highest (yellow) expression for each analyte; and (F,H) quantified as fold change induced by NID1. Nonparametric Wilcoxon Rank sum test. I,J) Protein ratios of (I) Bax to Bcl2 and (J) SAPK to Erk1,2 (*n* = 4); unpaired *t*‐test. K) Proposed common and separate mechanisms of action of NID1 for CMs and *β*‐cells in vitro. Middle: common pathway, NID1‐*α*v*β*3 ligation upregulating Fyn/Src in *β*‐cells and cdc42 in CMs, which stimulates Wnt3 and the MAPK pathway. Left: *β*‐cells, Fyn/Src activates the MAPK pathway, upregulating p21, which can be antiapoptotic. Fyn/Src crosstalks with Wnt3 and EpCAM, which enhances insulin secretion. Right: CMs, cdc42 downregulates the Hippo pathway as shown by an upregulation of MOB1. Pathways specific for each cell type are shown either on the left for *β*‐cells and on the right for CMs. *p < 0.05; **p < 0.01, ***p < 0.001, and ****p < 0.0001.

Understanding the mechanistic function of a therapeutic biomolecule is required before entering clinical trials. Here, we sought to elucidate the mechanisms driving the function of NID1 during hypoxic events. The integrin *α*v*β*3 has been reported as a binding partner for NID1 and is expressed on the cells of human Langerhans islets and on CMs.^[^ ^26^, ^27^, ^28^ ^]^ NID1 and *α*v*β*3 binding was confirmed at the protein level by titration of immobilized *α*v*β*3 with soluble NID1 (**Figure** **6**A). No fixation was employed, as this would have altered the protein binding. Coating of 4 µg mL^−1^ of *α*v*β*3 was sufficient to have a specific ELISA signal of bound NID1 in the presence of Mn^2+^ when compared with NID1 in EDTA, indicating a divalent cation‐dependent interaction of NID1 and *α*v*β*3 integrin. In addition, the binding was dose‐dependent as a specific ELISA signal was detected starting between 25 and 50 µg mL^−1^ of NID1 (corresponding to ≈0.167 × 10^−6^ m to 0.334 × 10^−6^ m).

Blocking of the *α*v*β*3 integrin on pseudoislets and hiPSC‐CMs was performed to investigate whether the positive effect of NID1 is mediated through the activation of the *α*v*β*3 integrin (Figure 6B–D). Blocking of NID1‐treated pseudoislets with an *α*v*β*3 antibody inhibited the increase in insulin secretion under normoxia (Figure 6B). Blocking *α*v*β*3 integrin in NID1‐treated hiPSC‐CMs under hypoxic conditions negated the decrease in apoptosis seen in nonblocked NID1‐treated hiPSC‐CMs in caspase‐3 staining and TUNEL assay (Figure 6C,D). These data indicate that *α*v*β*3 is involved in mediating the interaction of NID1 with *β*‐cells and CMs.

A novel high‐throughput digital Western blot platform (DigiWest) was employed to investigate the specific biological pathways that are mediated by NID1 in *β*‐cells and CMs cultured under hypoxic conditions.^[^ ^29^ ^]^ NID1‐treated *β*‐cells showed a significantly upregulated expression of EpCAM, Erk 2, pFyn, p21, Src, pSrc, Wnt3, and a trend toward the upregulation of pMEK1/2 (Figure 6E,F). NID1 treatment of hiPSC‐CMs led to an upregulation of Bax, pErk 1/2, SAPK, MOB1, Rac1/cdc42, and Wnt3 (Figure 6G,H) Downregulation was observed for b‐Raf, pFAK, and Notch2. Bax and SAPK protein contents were normalized by Bcl2 and Erk1,2 in order to determine whether proapoptotic pathways have been activated. Ratios of Bax/Bcl2 and SAPK/Erk1,2 showed no significant difference between NID1‐treated and control cultures (Figure 6I,J), which indicates that the increase in Bax and SAPK in the NID1‐treated hiPSC‐CMs had been offset by an increase in Bcl2 and Erk.^[^ ^30^, ^31^ ^]^ All target proteins tested and the resulting protein regulation are shown as heatmaps in Figure S7 of the Supporting Information. The hypothetical pathways associated with NID1 are proposed in Figure 6K. Here, we have shown that NID1 binds and signals through integrin *α*v*β*3 as seen by the upregulation of pFyn, Src, pSrc, and Rac1/cdc42. This leads to the activation of the mitogen‐activated protein kinases (MAPK) pathway, including the kinases extracellular signal‐regulated kinase 1/2 (Erk 1/2) and mitogen‐activated protein kinase 1/2, which is driving the protective effects of NID1 both on *β*‐cells and CMs.

## 3 Discussion

In this study, we report the protective and immune‐modulatory effects of NID1 on cells and tissues of the cardiovascular, pancreatic, and immune system. We demonstrate the therapeutic efficacy of NID1 by a significant improvement in heart function post‐MI/R in a preclinical mouse model and discovered a promising new avenue to improve the outcome of *β*‐cell transplantation. We propose that the effect of NID1 is mediated by the *α*v*β*3 integrin leading to the activation of the MAPK pathway in both CMs and *β*‐cells.

NID1 is an underinvestigated ECM BM protein that is mostly recognized as a linker protein of COL4 and LAM.^[^ ^32^ ^]^ It has been suggested to play a role in angiogenesis, hepatic regeneration, and regenerative axon growth and guidance.^[^ ^33^, ^34^, ^35^, ^36^ ^]^ Here, the identification of NID1 as the highest expressed BM component during cardiovascular differentiation, and the important role of BM proteins in organogenesis suggested that NID1 may have a regenerative or protective effect on the ischemic heart. The heart has a highly limited capacity for regeneration; therefore, current treatment strategies seek to restore cardiac function by methods such as cell injections into the infarct border zone, and delivering growth factors and small molecules or ECM proteins to activate or modify native cardiac cells.^[^ ^37^, ^38^ ^]^ Despite promising results, many of these approaches have questionable or unknown safety profiles, hindering their clinical translation. We propose a simplified strategy of one ECM protein that has a potential supportive effect on immune cells, as opposed to an inflammatory effect. The therapeutic ability of NID1 was demonstrated by an improved heart function in a post‐MI/R mouse model as demonstrated by elevated EF and FS, as well as the recovery of EDV, LVED, and LVIDd. Moreover, in vitro studies showed that NID1 suppresses the transdifferentiation of fibroblasts into fibrillar ECM‐secreting myofibroblasts, potentially enhancing the significant reduction of fibrosis seen in vivo. The ECM protein agrin was previously shown to improve heart function post‐MI through CM division and proliferation.^[^ ^39^ ^]^ Our findings are unique as we show that NID1 improves heart function by the protection of CMs and potential regeneration of other heart cells. Interestingly, both NID1 and agrin activate the MAPK pathway in CMs; however, NID1 ligates the *α*v*β*3 integrin and agrin ligates Dag1.

Our functional data suggest that NID1 is also applicable in other therapeutic applications where ischemia and fibrosis hinder therapeutic efficacy, such as in islet *β*‐cell transplantation. During the first week of islet transplantation, fibrosis encapsulates the transplant delivery device, and the lack of oxygen supply causes the loss of *β*‐cell mass, restricting the patient's insulin independency.^[^ ^40^ ^]^ Interestingly, NID1 was the only BM protein to have a specific spatial distribution that exclusively colocalized with insulin‐producing *β*‐cells. In vitro data showed that NID1 has a protective effect on *β*‐cells under hypoxic conditions and has a direct impact on insulin secretion in a dose‐dependent manner. This suggests that NID1 could be used as a protective and functional molecule during islet transplantation as it could enhance angiogenesis and reduces fibrosis around the device while improving *β*‐cell function and survival within.

We discovered that full length human NID1 binds to human integrin *α*v*β*3 in a divalent cation‐dependent way, and that *α*v*β*3 is the primary binding ligand driving the therapeutic effect of NID1 on CMs and *β*‐cells. The combination of gene and protein expression analysis confirmed the activation and downstream signaling of the *α*v*β*3 via the MAPK pathway in hiPSC‐CMs and *β*‐cells. MAPK effectors such as Erk 1/2 are expressed following oxidative stress in CMs, which protect them from apoptosis in vitro and in vivo.^[^ ^41^ ^]^ In addition, the MAPK pathway is essential for glucose‐stimulated insulin secretion in *β*‐cells.^[^ ^42^ ^]^ In CMs, an additional protective mechanism may be provided by shutting down the Hippo pathway, which has been described to inhibit adult cardiac regeneration.^[^ ^43^ ^]^ Wnt signaling, which may arise through *α*v*β*3 downstream signaling, is involved in *β*‐cell insulin secretion, as well as cellular survival in both cell types.^[^ ^44^, ^45^ ^]^ The MAPK and Wnt pathways as well as the stimulation of the *β*3 integrin are known activators of *TNC* expression.^[^ ^46^ ^]^ *TNC* is involved in CM detachment from the ECM that allows surviving CMs to reorganize and rebind to the ECM thus protecting CMs from anoikis.^[^ ^47^ ^]^ *TNC* and *THSP1* are known activators of TGF‐*β* signaling, which regulates the inflammatory response in the postinfarcted heart and the deposition of fibrous tissue.^[^ ^48^ ^]^ Taken together, our data uncovered the key mechanisms of NID1 action in vitro, providing insight into the therapeutic function of NID1 seen in vivo.

It is well established that the ECM has a modulatory function on a variety of immune cells. Monocytes migrate to injured tissue and polarize to distinct macrophage phenotypes depending on the surrounding tissue milieu.^[^ ^49^ ^]^ We identified in vitro a significant migration of CD14+ monocytes in the presence of NID1 and the influence of macrophage polarization toward a phenotype with characteristics of both the M0 and the regenerative M2 phenotype, but not the pro‐inflammatory M1 phenotype. It was previously shown that NID1 binds to natural killer cells via NKp44, mediating an inhibitory effect seen by the reduction of cytokine release such as IL‐2 and IFN*γ*.^[^ ^50^ ^]^ We demonstrated a similar reduction of proinflammatory cytokine secretion such as TNF*α* and IFN*γ* in activated T cells by NID1, indicating the anti‐inflammatory potential of NID1. Taken together with recent research showing that macrophages contribute collagen to scar formation, the NID1‐mediated modulation of macrophages may play an important role in the reduction of scar tissue seen in this study.^[^ ^51^ ^]^

## 4 Conclusion

In summary, in our study, in vitro and in vivo models demonstrated that NID1 increases cardiogenesis, angiogenesis, and cell survival, while beneficially modulating immune responses and reducing fibrosis. Mechanistically, NID1 was shown to ligate the *α*v*β*3 integrin to activate the Erk 1/2‐MAPK and Wnt pathways in cardiomyocytes and pancreatic *β*‐cells. In a preclinical mouse model, a single treatment with an NID1‐functionalized gel significantly increased heart function post‐MI. These data demonstrate the positive effect of NID1 on ischemic cells and tissues from different organs, suggesting that it may have multiple clinical applications as an off‐the‐shelf product.

## 5 Experimental Section

##### hESC Cultures and Cardiovascular Differentiation

The use of hESCs (H9, passages 36–60, WiCell) was approved by the Robert Koch‐Institute, Berlin, Germany (AZ: 3.04.02/0086). hESCs were maintained according to WiCell feeder‐dependent Pluripotent Stem Cell Protocols, SOP‐SH‐001, version G, on mouse embryonic fibroblasts (AMS Biotechnology (Europe) LTD) in stem cell growth medium. For EB generation, 3 × 10^4^ cells of an H9 single cell suspension in mTeSR1 (STEMCELL Technologies), supplemented with 10 ng mL^−1^ BMP4 and 10 ng mL^−1^ ROCK inhibitor Y‐27632 (Sigma‐Aldrich), were centrifuged per 96‐well (nontreated, conical bottom, Thermo Fisher Scientific) at 1000 rpm for 5 min and incubated overnight. Two previously described protocols were modified for cardiovascular differentiation.^[^ ^15^, ^16^ ^]^ On day 1, after the overnight incubation, up to 200 EBs were transferred to one ultralow attachment 6‐well well (Corning Inc.) in 3 mL of stage 1 media (StemPro 34 (Life Technologies), 3 ng mL^−1^ activin A, 5 ng mL^−1^ bFGF, 10 ng mL^−1^ BMP4). On day 4, the floating EBs were transferred to a 6‐well plate, coated with 0.1% gelatin or 0.1% gelatin‐supplemented with or without 50 µg mL^−1^ nidogen‐1 (NID1) and cultured in 2 mL^−1^ of stage 1 media + 5 × 10^−6^ m inhibitor of WNT response‐1 (IWR‐1). The EBs were incubated for 24 h to allow attachment. The attached EBs were differentiated in stage 2 media (StemPro 34, 5 ng mL^−1^ VEGF, 10 ng mL^−1^ bFGF, 5 × 10^−6^ m IWR‐1) from day 5 to day 10. Media changes were required every two days throughout cardiovascular differentiation. Attached EBs typically started beating on day 7 and were harvested for further analysis on day 10.

##### Ethics Information

This study was performed in accordance with institutional guidelines and was approved by the local research ethics committees (University of California, Los Angeles IRB #05‐10‐093; University Tübingen IRB #356‐2008BO2, #406‐2011BO1, and #495‐2018BO2; Landesärztekammer Baden‐Württemberg, IRB #F‐2012‐078 and #F‐2011‐068; and the Charité Universitätsmedizin Berlin #EA/226/14).

Human first trimester and second trimester tissues were obtained from electively aborted fetuses following informed consent and de‐identification. Normal adult heart sections were obtained from post‐mortem autopsy samples (expiration due to noncardiac cases, with no history or evidence of cardiac disease on post‐mortem inspection), which were provided by the department of pathology, David Geffen School of Medicine at UCLA, Los Angeles, USA.

All mouse surgeries were performed under the supervision and with approval of the UCLA Animal Review Committee (#2011‐042 and #2013‐057).

##### Histology, Histochemistry, Immunofluorescence Staining, and Quantification

Fetal tissues were freshly fixed in 4% PFA and embedded in paraffin using a Shandon Citadel 1000 (Thermo Fisher Scientific). The pseudoislets and EBs were fixed in 4% PFA, placed in Histogel (American MasterTech) and processed for paraffin embedding. All paraffin‐embedded cells and tissue were sectioned (3 µm sections) using a microtome HM340E (Thermo Fisher Scientific). Sections of adult pancreas (3 µm sections) were commercially obtained (NBP2‐30191, Novus Biologicals). Sections of adult human heart tissue were provided by UCLA as stated in the Ethics section.

Histological and histochemical staining was performed on deparaffinized and hydrated serial sections of explanted mouse hearts, 28 days post MI/R. Russell‐Movat pentachrome staining visualized collagens (yellow), muscle tissue (red), proteoglycans/ glycosaminoglycans (blue‐green), mature elastic fibers (black), and cell nuclei (dark red). Bright field (BF) images were acquired using a Zeiss Axio Observer Z1 (Carl Zeiss). Picrosirius Red and Fast Green stain visualized the collagen‐rich scar (red) and the myocardium (green). In detail, deparaffinized and hydrated sections were incubated for 5 min in 0.5% acidified water (acetic acid, Carl Roth) and subsequently for 1 h in a solution of 0.1% Fast Green FCF at pH 2 (Sigma‐Aldrich) and 0.1% Picrosirius Red in saturated picric acid (Morphisto) at a 1:1 ratio. The sections were cleaned twice in acidified water for 2 min, dehydrated using graded ethanol washes (70–100% v/v), and then mounted. High definition images were acquired with the glass slide scanner Opticlab H850 (Plusteck) and saved as TIFF. The glass scale bar 1972‐50 peak (GWJ Co) was used to scale the collected images. A MATLAB algorithm was developed to identify and quantify the image components based on their HSV histograms. Valves and vessels of the outflow tract were stained red and cropped out of the image to determine the true scar areas. All areas calculated were normalized to the total tissue area.

For immunofluorescence staining, the paraffin sections were stained as previously described.^[^ ^24^, ^25^, ^26^, ^27^, ^28^, ^29^, ^30^, ^31^, ^32^, ^33^, ^34^, ^35^, ^36^, ^37^, ^38^, ^39^, ^40^, ^41^, ^42^, ^43^, ^44^, ^45^, ^46^, ^47^, ^48^, ^49^, ^50^, ^51^, ^52^, ^53^ ^]^ Briefly, antigen retrieval was performed consecutively in Tris‐EDTA (pH = 9.0) and citrate buffer (pH = 6.0) in a steam cooker. For the intracellular antigens, the sections were treated with 1% Triton X‐100. A goat block solution was used to block unspecific binding sites. Antibodies were diluted in antibody dilution buffer (PBS containing 1% BSA, 0.1% TritonX‐100, 0.1% cold‐water Fish Skin Gelatin, 0.05% Tween20) and samples were incubated overnight at 4 °C. After several washes, the secondary antibody was applied to the samples and incubated for 30 min at room temperature, and after several washes the sections were exposed to a DAPI solution (5 µg mL^−1^ in PBS, Roche). Fluorescence images were acquired using a confocal laser scanning microscope (LSM 880 with Airyscan, Carl Zeiss Microscopy GmbH, Germany). The images acquired were processed with Adobe Photoshop CS5 (Adobe System Inc.). Semiquantitative analyses of IF images were conducted by detecting the gray value intensity (GVI) using ImageJ software. All GVI data were normalized to the laser power. Quantification of E‐cadherin and caspase‐3 staining of pseudoislets was conducted utilizing images obtained with a 63× magnification on a Zeiss Axio Observer Z1 (Carl Zeiss). The entire pseudoislet was selected as region of interest (ROI). GVI quantification of the ROI was conducted using ImageJ, version 1.52p. TUNEL assay was performed using the Click‐iT Alex Fluor Imaging Assay kit (Thermo Fisher Scientific). Sections were mounted with Prolong Gold Anti Fade solution (Thermo Fisher Scientific). TUNEL images were evaluated in a double‐blinded study by two unbiased observers. Cells were identified as TUNEL^+^ when a clear green and blue staining was exhibited. The ratio was calculated by dividing TUNEL^+^ cells by total number of DAPI^+^ cells per pseudoislet. Images were obtained using a 63× magnification on a Zeiss Axio Observer Z1 (Carl Zeiss). Quantification of the vessel density in *α*SMA/CD31/CTNT‐stained mouse infarct scar area sections was conducted by counting *α*SMA+/CD31+ vessels utilizing images taken with a 25× objective (*n* = 6). The scar area of each image was measured using the ImageJ software. To quantify colocalization, a macro was designed using Microsoft Excel, version Office 365. Briefly, the algorithm compares GVI values of pixels of the simultaneously obtained NID1 and insulin channels. Colocalization was counted when the GVI of a specific pixel exceeded a set threshold on both channels and normalized by the total amount of pixels exceeding the threshold value in the NID1 channel. TuJ1 was used for the assessment of neuronal cells in the infarct area. 10 IF images per heart were taken with a 63× objective. TuJ1^+^ and total cells were counted.

##### NID1 Production Plasmid

The inducible NID1 production plasmid was constructed from several sections of commercially available vectors. The main backbone consisted of the pcDNA3.1 vector. The inducible TRE promotor for NID1 expression was taken from the pTRE3G vector and DHFR expression was controlled by the TK promoter from the pGL4.74(hRluc/TK) vector. A codon‐optimized sequence of human NID1 (GenBank accession number: BC045606.1, 5406 bp) with a histidine/asparagine tag at the C‐terminus was synthesized from GeneArt (Life Technologies).

##### Stable NID1 Production Clone Generation and Production Induction

CHO DHFR‐negative mutant cells (DSMZ no.: ACC 126) were transfected with the pCMV‐Tet3G plasmid (631166, Clontech Laboratories) and selected for Geneticin (G418, Calbiochem) resistance. Selected activator clones were transfected with the NID1 production plasmid and cultured for two weeks in selection media (MEM *α*, 10% FBS dialyzed, 500 µg mL^−1^ of G418), which does not support the growth of DHFR‐negative cells. Single cell cloning was conducted and clones were treated with 100 ng mL^−1^ doxycycline hydrochloride (Dox) (Thermo Fisher Scientific) to induce NID1 production. Secreted NID1 was measured using an ELISA (DY2570, R&D Systems). Clones with the highest measured levels of NID1 underwent selection rounds with rising levels of methotrexate (MTX) for genomic amplification. During MTX selection, ELISA, q‐RT‐PCR, and q‐PCR were performed to measure NID1 protein levels and assess the relative levels of NID1 mRNA and the relative copy number of genome amplified NID1. The best production clones were adapted to suspension growth in Erlenmeyer flasks with serum‐reduced media (DMEM/Ham's F12 basal medium, 0.5% FBS dialyzed, 2.5 × 10^−6^ m MTX, 2 × 10^−3^ m l‐Glutamine, 1% penicillin–streptomycin (Pen/Strep), 250 µg mL^−1^ G418) in an incubation shaker (Minitron, Infors GmbH) at 37 °C and 5% CO_2_ at 85 rpm. NID1 expression was induced with 100 ng mL^−1^ Dox and the media with secreted NID1 was harvested 4–5 days later and stored before NID1 purification at −20 °C.

##### NID1 Purification

The IMAC purification of the histidine/asparagine‐tagged human NID1 was performed with a HisPrep FF 16/10 affinity chromatography column (GE Healthcare), controlled by the FPLC system Äkta Explorer 10 (GE Healthcare). NID1 elution was indicated by the increased absorption at the wavelengths 280 and 256 nm. Protein‐containing elution fractions were pooled and desalted using a HiPrep 26/10 desalting column (GE Healthcare), controlled by the Äkta Purifier 100 (GE Healthcare). Subsequently, the protein solution was washed and concentrated with ultrafiltration units (Vivaspin 20, Sartorius), sterile filtered (SCGP00525, Millipore), and stored at −80 °C until further use.

##### SDS‐PAGE and Western Blot

All protein samples were mixed with 4× Roti‐Load (Carl Roth) and denatured at 90 °C for 5 min. The samples were run on a NuPAGE Novex 3–8% Tris‐Acetate gel (EA03752BOX, Life Technologies) under denaturing conditions. The HiMark Pre‐Stained Protein Standard was used for easy band identification. After SDS‐PAGE, the proteins were transferred to a nitrocellulose membrane (Whatman) in an electrical field (30 V, 60 min) in the XCell II Blot Module (Life Technologies). For an unspecific protein staining, the nitrocellulose membrane was incubated in a Ponceau‐Red solution (Sigma‐Aldrich) for 5 min at room temperature. Images were acquired and the membrane was discolored in a 0.1 m NaOH solution for later specific protein immunodetection. The membrane was blocked using 5% skim milk powder (Sigma‐Aldrich) in TBS‐T and then incubated with the primary antibodies overnight at 4 °C while shaking. After several washes with TBS‐T, the membrane was incubated with the secondary antibody in TBS‐T with 5% skim milk powder for 1 h at room temperature. After washing with TBS‐T, SuperSignal West Dura Extended Duration Substrate (Thermo Fisher Scientific) was applied onto the membrane, drained after few seconds and the developed chemiluminescence was detected in the Luminescent Image Analyzer LAS‐1000 plus (FujiFilm).

##### NID1 Deglycosylation

Purified NID1 was deglycosylated with an enzyme mix (P6039S, New England Biolabs) as instructed by the manufacturer. In addition to the 4 h incubation time described by the manufacturer, an identical reaction mix was incubated for 20 h. The control sample was treated accordingly, except for the addition of the deglycosylation enzymes, and incubated for 4 h. NID1 deglycosylation was analyzed by the mobility shift of the NID1 bands to a lower protein size on the SDS‐PAGE gel.

##### Co‐Immunoprecipitation (Co‐IP)

The protocol for Co‐IP was modified from previously published studies.^[^ ^54^, ^55^ ^]^ The interaction partners NID1 (1 µg) and LAM 511 (0.5 µg, LN511‐02, BioLamina) were mixed in 500 µL of a dilution and washing buffer (0.1 m NaCI, 0.05 m Tris/HCl pH 7.4 containing 0.04% Tween‐20 and 1% BSA) and were agitated gently on a rotisserie mixer overnight. 6 µg of the antibody against LAM (ab11575, Abcam) was added to the protein mix and incubated for 24 h for Co‐IP. This antibody was not added in the negative control (unspecific background control). Protein A magnetic beads (LSKMAGA02, Millipore) were added and gently agitated on the rotisserie mixer with the protein complexes ± the LAM antibody for 2.5 h at 4 °C and another 30 min at room temperature. After washing off the magnetic beads using a magnetic stand (LSKMAGS08, Millipore), the protein complexes were eluted from the beads and denatured in 30 µL of 1× Roti‐Load (K929.2, Carl Roth) via heating for 10 min at 90 °C. Detection of the specific protein bands occurred after SDS‐PAGE and Western blot.

##### qPCR and Expression Profiling Using RT2 Profiler PCR Array

RNA extraction of dermal fibroblasts was performed on lysed cells. Briefly, RNA was isolated using sequential incubation and centrifugation of the lysate with chloroform (Sigma‐Aldrich), isopropanol (Carl Roth), and 75% ethanol (Applichem). RNA was resuspended in nuclease free‐water and heated for 10 min at 55 °C prior to RNA quantification. RNA was reverse transcribed according to the manufacturer's instructions (RNeasy Micro Kit, Omniscript RT Kit, Qiagen). qPCR of EBs was performed on the LightCycler 480 II (Roche) with the 480 SYBR Green I Master (04887352001, Roche) for the primers NID1, p53 and *β*‐actin for normalization and reference as listed in Extended Data in Table S4 of the Supporting Information. qPCR was performed using a Bio‐Rad CFX96 system and the QuantiFast SYBR Green PCR Kit (Qiagen) for the primers *TNNT2* (QT00089782, Qiagen) and *ACTA2* (QT00088102, Qiagen).

qPCR on dermal fibroblasts was performed using ACTA2 (QT00088102, Qiagen), GAPDH (QT01192646, Qiagen) and the QuantiNova SYBR green PCR Kit (208052, Qiagen). All samples were performed as triplicates. The 2^−∆∆Ct^ method was applied for qPCR data quantification.

For expression profiling using RT^2^ profiler PCR Array (Qiagen), RNA extraction was performed according to manufacturer's instructions (RNeasy Micro Kit, Omniscript RT Kit, Qiagen). RNA was reverse transcribed to cDNA using RT2 First Strand Kit (SABiosciences, Qiagen). The Human Extracellular Matrix & Adhesion Molecules RT^2^ Profiler PCR Array (PAHS‐013ZD; Qiagen) was used to measure expression levels of 84 individual genes important for cell–cell and cell–matrix interactions. For all RNA and DNA quantifications, absorbance was measured using a NanoQuant Plate in combination with Infinite 200 PRO (Tecan). If not mentioned otherwise, qPCR measurements were performed using a Bio‐Rad CFX96 system and data analysis was performed using the Qiagen data analysis center online tool.

##### Imaging Flow Cytometry

EBs were harvested at day 10 of the cardiovascular differentiation protocol to conduct flow cytometry analyses using a standard staining protocol.^[^ ^56^ ^]^ Briefly, cells were incubated with Zombie Red dye (BioLegend) to exclude dead cells and CD29‐PE (MCA2298PE, AbD Serotec, UK) for 25 min at 4 °C protected from light. After washing, the Foxp3 Staining Buffer Kit (421403, BioLegend) was used to fix the cells for 30 min on ice, followed by washing and permeabilization for 15 min at 4 °C. After blocking with BSA for 10 min, cells were incubated with CTNT primary antibody (ab8295, Abcam) for 35 min at 4 °C, washed and blocked with 5% BSA for 10 min at room temperature to avoid unspecific binding. The secondary anitbody (rat antimouse IgG1‐AF647, 406617, BioLegend) was added for 25 min at 4 °C, protected from light. For the analysis using the ImageStreamx mkII (Amnis Corporation, USA) with the INSPIRE instrument controller software with 40× magnification, 1 × 10^4^ single cells were acquired per sample. Data were analyzed with IDEAS Image analysis software. All samples were gated on single cells that were Zombie Red‐negative. The percentage of CTNT+ cells was determined and a control sample (stained with the same antibodies except the primary antibody) was used to set the background fluorescence.

##### In Vivo Mouse MI/R Model and NID1 Injections

A mouse MI/R model was used to identify the effect of myocardial NID1 injections post MI.^[^ ^19^ ^]^ All surgeries were performed at UCLA. In detail, 8 weeks old female C57BL/6J mice, strain DLAMb6, were anesthetized using 2.0% isoflurane (Butler Schein), placed on a heated surgical board and given 2.5 mg kg^−1^ flunixin (Flunixin Meglumine, Schering‐Plough Animal Health) subcutaneously. Under a dissecting microscope, a midline cervical incision was made to expose the trachea for intubation with a PE‐90 plastic catheter (Stoelting Company). The catheter was connected to a Harvard minivent (Harvard Apparatus) supplying oxygen with a tide volume of 225–250 µL and a respiratory rate of 130 strokes per minute. Surgical plane anesthesia was subsequently maintained with 1–1.5% isoflurane. A lateral incision was made in the fourth intercostal space. The heart was exposed and the left anterior descending coronary artery (LAD) was ligated intramurally 2 mm from its origin with a 9‐0 nylon suture (Ethicon). The suture was tied around a small piece of plastic tubing (PE‐10) to occlude the coronary artery while allowing an easier and safer relief of the occlusion. This occlusion occurred for 45 min. During this time, a moist gauze pad was placed over the incision to maintain a sterile atmosphere. Ischemia was verified by the regional paleness of the myocardium that was no longer supplied with blood by the left coronary artery. Reperfusion was allowed by cutting the knot on the PE‐10 tube and could be verified by the appearance of hyperaemia in the previously pale region. Reperfusion caused the infarcted region to change into a normal pinkish red color. This procedure was conducted on five mice for the NID + HA treatment group, five mice for the HA carrier gel group, and three mice for the saline control group. Samples sizes were determined for statistical power and in accordance to the EU 3Rs regulation for the reduction of animal studies. After confirming that there was no bleeding, the animals received injections in the MI border zone. Three animals in the control group were injected with 50 µL of saline solution. Five animals were injected with a total of 50 µL of HyStem hyaluronic acid (HA) hydrogel (Glycosan BioSystems) in PBS per animal. In the NID1 + HA group, five animals received 50 µL injections of HA in PBS with 50 µg mL^−1^ NID1. For each mouse, the 50 µL total injection volume was divided into 10 µL injections at five sites in the LV myocardial infarction border zone. The mouse chests were closed in two layers. The ribs (inner layer) were closed with 6‐0 coated vicryl sutures (Ethicon) in an interrupted pattern. The skin was closed using 6‐0 nylon or silk sutures (Covidien) in a subcuticular manner. The anesthesia was stopped and the mice were allowed to recover for several minutes before the endotracheal tube was removed. The mice received intraperitoneal injections of 2.5 mg kg^−1^ Banamine postsurgery for the alleviation of pain. One mouse in the NID1 + HA treatment group died of arrhythmia after surgery, which lead to the total number of four animals in this group. Two animals from the HA carrier gel group (HA controls) had to be excluded due to incomplete ischemia, giving the final number of three for this group.

##### Hydrogel Preparation with and Without Supplementation of NID1

All procedures for control and NID1 + HA injections were conducted under sterile conditions. The HA hydrogel was prepared according to the manufacturer's instructions (HyStem) and was further diluted to 2:13. For each mouse, a total of 50 µL HA in PBS ± 50 µg mL^−1^ NID1 was needed; therefore, due to possible residues in the preparation tube or in the syringe, 70 µL were prepared per mouse (HA: PBS ± 50 µg mL^−1^ NID1).

##### Echocardiography

Conscious echocardiography was conducted on three different time points. A baseline echocardiography measurement was conducted on each mouse prior to the MI/R procedure. In serial nature, further echocardiography measurements were conducted 2 days and 28 days after the MI/R and myocardial injection procedure to track cardiac function. For noninvasive echocardiographic imaging of the conscious mice in vivo, the high‐frequency ultrasound imaging system Vevo 2100 (VisualSonics) was applied with the MS400 transducer 18–38 MHz. Myocardial performance was assessed via the Vevo LAB software. The mice were sacrificed after the 28‐day echocardiography, and their hearts were explanted for histological and immunofluorescence assessment.

##### Raman Imaging of Mouse Heart Sections and Pseudoislets

Islets were prepared as previously described.^[^ ^24^ ^]^ Briefly, hypoxic NID1‐treated and control pseudoislets were placed in a microfluidic chip for noninvasive in situ Raman imaging as previously performed.^[^ ^24^, ^57^ ^]^ Spectral mapping was performed on a customized inverted WITec Raman system (WITec GmbH) equipped with a green laser (532 nm) and a CCD spectrograph with a grating of 600 g mm^−1^. Images were acquired at a laser power of 50 mW, an integration time per spectrum of 0.5 s, a pixel resolution of 1 × 1 µm and at least as triplicates. Based on Movat staining, representative sections were selected from each heart and deparaffinized. The sections were kept at 4 °C in PBS until measurement. The spectra were collected with a 63× dipping objective (W Plan‐apochromat; Carl Zeiss), a laser power of 60 mW and an integration time of 0.05 s (per pixel). In order to locate the scar, an overview image of 1000 × 1700 µm was collected from the apex of the heart with 1 µm image resolution. Within this scan, 3 ROIs of 200 × 200 µm were acquired with a resolution of 0.5 µm.

##### Multivariate Data Analysis

Raman images were processed and analyzed by TCA using the Project FIVE 5.2 software (WITec GmbH).^[^ ^23^ ^]^ Briefly, TCA is a non‐negative matrix factorization‐based MVA tool that elaborates spectral components that predominantly occur in the data set and allows the identification and localization of these components by false color intensity distribution heatmaps. GVI per pixel were determined in ImageJ to semiquantify the distribution of the spectral components in both conditions. Furthermore, TCA allowed the preselection of ROIs of similar spectral information, which were extracted for further in‐depth analysis of the molecular composition by PCA using Unscrambler X (Camo). Moreover, MCR analysis was applied to define the local spectral composition within the islet periphery and islet core of the pseudoislets. NID1 core, control core, NID1 periphery and control periphery were defined by the islet area and compared to determine the protective effect of NID1 on the pseudoislets.

##### hiPSC‐Derived Cardiomyocyte Cell Culture

iCell Cardiomyocytes2 (cat: #R1057, Cellular Dynamics) culture was performed according to the manufacturer's specifications. Briefly, 96‐well plates were coated with 0.1% porcine gelatin solution for 1 h. hiPSC‐CMs were seeded at a density of 2 × 10^4^ cells mL^−1^. At day 9, hiPSC‐CMs were treated with 50 µg mL^−1^ NID1 and placed in normoxic or hypoxic conditions for 48 h. At day 9, hiPSC‐CMs were treated with 50 µg mL^−1^ NID1, or PBS as control, and placed in normoxic or hypoxic conditions for 48 h. Cells were either fixed in 4% PFA for IF staining, lysed in TRI‐Reagent (Sigma‐Aldrich) for RNA isolation and qPCR or prepared for DigiWest evaluation. For DigiWest evaluation, the cells were detached using TryplE (Gibco), washed five times on ice and snap frozen in liquid nitrogen.

##### Fibroblast Cultures

Fibroblasts were isolated from adult skin and myocardial biopsies after informed consent was given (Landesärztekammer Baden‐Württemberg, IRB #F‐2012‐078 and #F‐2011‐068) as previously reported.^[^ ^58^, ^59^ ^]^ Fibroblasts were cultured until 90% confluency. Prior to seeding, 3.5 mm dishes (glass bottom µ‐Dish, ibidi) were coated with 0.1% porcine gelatin coating (Sigma‐Aldrich) for 1 h. 1 × 10^5^ cells per dish were seeded in media containing 50 µg mL^−1^ NID1 and placed in normoxic or hypoxic conditions for 48 h. After incubation, cells were either fixed in 4% PFA for IF staining or lysed in TRI‐Reagent (Sigma‐Aldrich) for RNA isolation and qPCR.

##### Tube Formation Assay

HUVECs (single donor cells, C2517A, Lonza) were thawed and cultured in EGM‐2 medium per manufacturer's instructions (CC‐4176, Lonza) at 37 °C and 5% CO_2_. One day before the tube formation assay, the cells were equilibrated in low‐serum EGM2 medium (0.5% FBS and no other supplements). HUVECs were seeded in a cell density of 1.5 × 10^4^ cells/96‐well well (1.5 × 10^4^ cells/ 0.32 cm^2^) in triplicates on serum‐reduced Matrigel only, or serum‐reduced Matrigel supplemented with 50 µg mL^−1^ NID1. All materials for the coating were precooled and the coating was performed with 75 µL per 0.32 cm^2^. Following 75 µL low serum EGM‐2 medium, 100 µg mL^−1^ NID1 were added to the respective wells and allowed to equilibrate at 37 °C and 5% CO_2_ for 30 min. 75 µL of the cell suspension was added per 96‐well and incubated for 2 h. HUVEC tubular structure formation was assessed with a Zeiss Axio Observer Z1 (Carl Zeiss). Quantification of different tube formation parameters was performed using the angiogenesis analyzer of ImageJ Version 1.50 g, which allows for the automated visual recognition of different tube formation parameters.

##### EndoC‐*β*h3 Cell Culture and Pseudoislet Formation

The conditionally immortalized human pancreatic beta cell line EndoC‐ßH3 was cultured per manufacturer's instructions. After 21 days, pseudoislets were formed as previously described.^[^ ^24^ ^]^ After 48 h in standard culture, pseudoislets were incubated for an additional 72 h under hypoxic (37 °C, 5% CO_2_, and 1% O_2_) or normoxic (37 °C, 5% CO_2_, and 21% O_2_) conditions. NID1 was supplemented in the media 72 h after seeding and for an additional 48 h at 20, 30, and 40 µg mL^−1^. PBS was used as negative control. After 72 h, pseudoislets were either subjected to a glucose‐stimulated insulin secretion (GSIS) assay, fixed in 4% PFA (Sigma‐Aldrich) for IF staining or prepared for DigiWest evaluation. For DigiWest evaluation, pseudoislets were grouped and washed five times on ice, centrifuged at 700 x *g* and snap frozen in liquid nitrogen.

##### Pseudoislet Size Tracking

A minimum of 10 pseudoislets were imaged every 24 h with a brightfield microscope (Carl Zeiss). Images were further processed with the ImageJ software, version 1.52p, where the average area was evaluated and subsequent average diameter calculated.

##### GSIS Assays

Prior to all GSIS assays, pseudoislets were incubated with NID1‐ or PBS‐ OPTI*β*2 (Univercell Biosolutions) medium for 24 h in normoxic or hypoxic condition. GSIS assays were performed after 5 days as previously described, except otherwise mentioned.^[^ ^23^, ^60^ ^]^ Pseudoislets were grouped by 6 to 8 per well and washed twice with *β*‐Krebs (Univercell Biosolutions) and 1% BSA (Thermo Fisher Scientific). Pseudoislets were preincubated for 1 h with Krebs‐BSA and the supernatant was discarded. 20 × 10^−3^ m glucose (Thermo Fisher Scientific) solution was prepared in Krebs‐BSA, and pseudoislets were incubated with either Krebs‐BSA or 20 × 10^−3^ m glucose solution for 1 h. The supernatant was collected and stored at −20 °C. An ultrasensitive insulin ELISA (Mercodia) was performed and the GSIS index was calculated by dividing the amount of secreted insulin during high glucose treatment by the amount of secreted insulin during basal state.

##### NID1 Cytotoxicity Test

A standardized cytotoxicity test according to EN ISO 10993 was performed using primary isolated human dermal fibroblasts (IRB #F‐2012‐078). An additional cytotoxicity test was performed utilizing primary isolated human cardiac fibroblasts (IRB #F‐2011‐068). 2 × 10^4^ cells were seeded per 96‐well well. In parallel, sterile solutions containing 50, 100, and 200 µg mL^−1^ NID1 were prepared in Dulbecco's modified Eagle medium (DMEM, Life Technologies). After 24 h, 200 µL of the NID1 solution was pipetted into each well and incubated for another 24 h. The cytotoxic substance SDS, which induces cell death, served as the positive control, and DMEM was used as the negative control. After incubation, the medium was removed and an MTS assay was performed. The cells of each well were incubated with a solution of 20 µL MTS (CellTiter 96 AQueous One Solution Cell Proliferation Assay, Promega) mixed with 100 µL DMEM. The absorbance was measured at 492 nm (Infinite 200 PRO, Tecan) after 45 min and the percentage of viable cells was calculated relative to the negative control, which was set to 100%. In all samples, cell numbers were counted.

##### Isolation of Human Immune Cells

Peripheral blood mononuclear cells (PBMCs) were isolated from buffy coats (DRK, Berlin, Germany, IRB #EA/226/14) as previously described.^[^ ^61^ ^]^ Briefly, blood was diluted (1:2) with PBS (Biochrom, Germany), layered on Biocoll Separation Solution (Biochrom) and centrifuged for 30 min at 800 x *g* at room temperature. PBMCs were isolated and washed three times with PBS. PBMCs were used to isolate CD14^+^ monocytic cells by CD14 MicroBeads (Miltenyi Biotec) according to manufacturer's instructions with MACS separation columns (Miltenyi Biotec). Labeling with CD14 PerCP/Cy5.5 (BioLegend) confirmed a purity of 95–98%.

##### Cultures of Human Macrophages with NID1

1 × 10^6^ monocytes per well or 7 × 10^5^ M0‐macrophages per well were seeded in 24‐well culture plates and were treated with NID1 at 50 µg mL^−1^ in VLE‐RPMI (Biochrom) containing 10% AB‐Serum (Sigma‐Aldrich) 1% penicillin/streptomycin (Life Technologies) and 1% glutamine (Life Technologies) at 37 °C in a 5% CO2 incubator for 7 days or 24 h, respectively. Monocytes (2 × 10^6^ mL^−1^) were differentiated into M0‐type macrophages for 7 days in 6‐well culture plates using 50 ng mL^−1^ M‐CSF (Miltenyi Biotec) in complete VLE‐RPMI. M0‐type macrophages were polarized for 24 h either toward M1 macrophages by adding 20 ng mL^−1^ IFN‐*γ* (Miltenyi Biotec) and 100 ng mL^−1^ LPS from *E. coli* O127:B4 (Sigma‐Aldrich) or into M2 macrophages by adding 20 ng mL^−1^ IL‐4 (Miltenyi Biotec) according to the recently described method.^[^ ^54^ ^]^ Representative images were obtained using a brightfield microscope (Carl Zeiss).

##### Macrophage Flow Cytometry Analyses

As described above, macrophages were harvested using 1% (v/v) trypsin/EDTA (Life Technologies), washed with PBS and labeled with a master mix of fluorophore‐labeled human‐specific antibodies CD163‐FITC, CD80‐PE, HLA‐DR‐PE/Cy7, CD206‐APC (all BioLegend), CD14‐APC/Cy7 (BD Biosciences), and a Live/Dead violet fixable staining kit (Molecular Probes).^[^ ^54^, ^61^ ^]^ Samples were measured with FACS Canto II (BD Biosciences) and analyzed using FlowJo Version 8.8.6 (TreeStar Inc.). Surface marker expression levels were normalized to the unstimulated controls (set as 1).

##### Immune Cell Proliferation Assay

Modulation of NID1‐treated T cell subset proliferation was analyzed with a CFSE‐based proliferation assay as described previously.^[^ ^24^ ^]^ Briefly, 96‐well culture plates were coated with 0.05 µg mL^−1^ anti‐CD3 antibody (OKT3, Janssen‐Cillag) overnight at 4 °C. Wells were washed three times with PBS, coated with 50 µg mL^−1^ NID1 for 6 h at room temperature and washed again with PBS. Isolated PBMCs were labeled with 2.5 × 10^−6^ m 5.6‐CFDA‐SE and seeded at a cell density of 3 × 10^5^ cells per well in complete VLE‐RPMI in wells coated with anti‐CD3 and NID1, anti‐CD3 alone (positive control) or they were used uncoated (unstimulated control). After 5 days of culture at 37 °C and 5% CO_2_, PBMCs were harvested and labeled with human‐specific antibodies CD8‐PE and CD4‐APC (both Miltenyi Biotec) as well as CD3‐APC/Cy7 (BioLegend) and Live/Dead violet fixable staining kit. Samples were processed using a FACS Canto II and data were analyzed using FlowJo Version 8.8.6. MFI of all markers were normalized to the anti‐CD3 control MFI level (set to 1).

##### Cytokine Detection Assays

Supernatants of NID1‐treated macrophage cultures (24 h or 5 days) and NID1‐treated PBMC proliferation assays (5 days) were collected for detection of released cytokines IFN*γ*, TNF*α*, IL‐6, and IL‐10 using ELISA kits (BioLegend) according to the manufacturer's instructions. Samples were measured on a Micro‐plate reader (Bio‐Rad).

##### Monocyte Chemotaxis Assay

To analyze chemotaxis, a specific 96‐well cell migration system (Neuro Probe) was applied as previously described.^[^ ^54^ ^]^ Briefly, the chemotactic stimulus was added in a volume of 37.5 µL per well. A membrane filter with 5 µm pores was placed onto the plate. 3 × 10^4^ cells in 40 µL diet medium consisting of VLE‐RPMI (Biochrom) and 0.1% autologous serum were added on the membrane. Chemotaxis was induced by 50 or 100 µg mL^−1^ NID1 or without stimulus (negative control). MCP‐1 (50 ng mL^−1^, Miltenyi Biotec) served as positive control. After incubation for 3 h at 37 °C in a 5% CO_2_ incubator, the membrane was carefully removed. Monocytes that adhered to the membrane were fixed with methanol (Merck) and labeled with Hemacolor staining kit (Merck). After microscopic documentation using ProgRes CapturePro 2.8.8 (Jenoptik), the number of migrated monocytes was determined using ImageJ Version 1.4.3.67 (National Institutes of Health). The number of migrated cells was normalized to the negative control.

##### Endotoxin Test

CD14^+^ monocytes (1 × 10^6^) were incubated alone (negative control), with 100 ng mL^−1^ LPS O127:B4 (Sigma‐Aldrich, positive control) or with 50 µg mL^−1^ NID1 in 24‐well plates in 1 mL^−1^ of VLE‐RPMI (Biochrom). After 24 h, the supernatant was collected and tested for TNF*α* by ELISA (BioLegend). In addition, a Pierce Chromogenic LAL Endotoxin quantification kit (Thermo Fisher Scientific) was employed according to the manufacturer's instructions.

##### Integrin Binding Assays

Binding assays were performed as previously described.^[^ ^62^ ^]^ Microtiter plates (Costar Half Area plate) were coated overnight at 4 °C with 50 μLper well and 4 µg mL^−1^ *α*v*β*3 (R&D Systems) in TBS containing 2 × 10^−3^ m MgCl_2_ or TBS with 2 × 10^−3^ m MgCl_2_ and 1 × 10^−3^ m CaCl_2,_ respectively. Plates were washed twice with the respective buffers. Blocking of nonspecific binding was performed for 1 h at room temperature with 1% BSA/TBS/MgCl_2_ or 1% BSA/TBS/ MgCl_2_/CaCl_2_. Serial dilutions of NID1 were prepared on ice. For *α*v*β*3, NID1 serial dilutions were prepared in 1% BSA/TBS/MgCl_2_ and 1 × 10^−3^ m MnCl_2_, or in 1% BSA/TBS/MgCl_2_ and 10 × 10^−3^ m EDTA‐buffer for nonspecific binding. Reaction was stopped using 1.5 m NaOH solution. OD determination was performed at 405 nm. Three independent experiments were performed, each of them with duplicates.

##### Blocking of Integrin *α*v*β*3

Mouse IgG (Vector Laboratories) and *α*v*β*3 (Novus Biologicals) were used at a concentration of 10 µg mL^−1^ in PBS. Pseudoislets at 48 h and CMs at 9 days, respectively, were washed twice with PBS and incubated with the blocking antibody or control for 1 h under standard culture conditions. Pseudoislets and CMs were washed twice with PBS. The respective NID1‐treated culture media or control were added to the pseudoislet or CM culture. Pseudoislets were culture for an additional 72 h and CMs for an additional 48 h before evaluation via IF staining or GSIS assay, respectively.

##### DigiWest

DigiWest assays (NMI Reutlingen, Germany) were performed as recently described.^[^ ^30^ ^]^ Briefly, gel electrophoresis and blotting onto PVDF membranes was performed using the NuPAGE system as recommended by the manufacturer (Life Technologies, Carlsbad, CA, USA). Blots were washed in PBST, proteins were biotinylated on the membranes using NHS‐PEG12‐Biotin (50 × 10^−6^ m) in PBST for 1 h followed by washing in PBST and drying. Individual sample lanes were cut into 96 molecular weight fractions (0.5 mm each) and proteins were eluted in 96‐well plates using 10 µL elution buffer per well (8 m urea, 1% Triton‐X100 in 100 × 10^−3^ m Tris‐HCl pH 9.5). Eluted proteins from each molecular weight fraction were loaded onto color‐coded, neutravidin coated Luminex bead sets (MagPlex, Luminex, Austin, TX, USA). 384 Luminex bead sets were employed and the protein loaded beads from four different sample lanes were pooled into one bead‐mix resulting in 6 bead mixes for the 24 samples. The bead‐mixes were sufficient for 100 antibody incubations (see Extended Data in Table S5, Supporting Information). Aliquots of the DigiWest bead‐mixes (1/100th per well) were added to 96‐well plates containing 50 µL per well assay buffer (Blocking Reagent for ELISA supplemented with 0.2% milk powder, 0.05% Tween‐20, and 0.02% sodium azide, Roche). Beads were briefly incubated in assay buffer and the buffer was discarded. Antibodies were diluted in an assay buffer and 30 µL were added per well. After overnight incubation at 15 °C on a shaker, the bead‐mixes were washed twice with PBST and PE‐labeled (Phycoerythrin) secondary antibodies (Dianova) were added and incubated for 1 h at 23 °C. Beads were washed twice prior to readout on a Luminex FlexMAP 3D. Secondary antibodies were either diluted in an assay buffer or in a polymer buffer (Blocking Reagent for ELISA supplemented with 4% PVP 360.000, 1% milk powder, 0.05% Tween‐20, and 0.02% sodium azide, Roche). For quantification of the antibody specific signals the DigiWest analysis tool (version 3.8.6.1, Excel‐based) was employed. This tool uses the 96 values for each initial lane obtained from the Luminex measurements on the 96 molecular weight fractions, identifies the peaks at the appropriate molecular weight, calculates a baseline using the local background and integrates the peaks. The values are based on relative fluorescence (accumulated fluorescence intensity). For analysis, data (measured signal intensity) was normalized to the total protein amount corresponding to the sample, median centered and log_2_‐transformed. The software package MEV 4.8.1 was used for data visualization, clustering, and nonparametric statistical analysis.

##### Statistical Analysis

The relevant statistical tests, sample sizes, replicate types, and *p*‐values are described in the corresponding figures and tables of the Supporting Information. Each *n* represents a distinct sample. Statistical tests were two‐sided if not mentioned otherwise. Data are presented as mean ± SD and as dot plots every time possible. All data sets were tested for normality. Statistical analysis was performed using Prism, version 6.07 (GraphPad Software). Statistical significance was defined at *p* < 0.05.

## Conflict of Interest

A.Z., S.L.L., G.P.D., and K.S.‐L. are inventors on patent application EP19154849.4 associated with this work and owned by the University Tübingen. S.L.L., M.Z., and K.S.‐L. are inventors on patents (EP3027201B1 and CN105517564A), and patent applications (US20160158314A1, CA2916614A1, JP2016530532A, and KR20160037170A) associated with this work and owned by the NMI, Reutlingen.

## Author Contributions

A.Z. and S.L.L. contributed equally to this work. A.Z., S.L.L., M.U., M.Z., H.K.A.M., A.N., M.S., and K.S.‐L. designed the experiments and wrote the manuscript. A.Z., S.L.L., M.U., D.A.C.B., J.M., M.Z., A.H., E.B., K.S., S.F., M.T., S.L., J.A.E., and M.S. performed experiments, collected and analyzed data. G.M.C., A.D., G.P.D., and G.K. provided reagents and gave conceptual advice.

## Supporting information

Supporting Information
