# Supplementary material for: Nidogen‐1 Mitigates Ischemia and Promotes Tissue Survival and Regeneration
Source: Adv Sci (Weinh). 2020 Dec 21;8(4):2002500. doi: 10.1002/advs.202002500 (PMC7887579; doi:10.1002/advs.202002500)
Supplement: Supplementary file 1 — Supporting Information [file ADVS-8-2002500-s001.pdf]

WILEY-VCH

© 2020 Wiley-VCH GmbH

ADVANCED SCIENCE
Open Access

Supporting Information

for *Adv. Sci.*, DOI: 10.1002/advs.202002500

Nidogen-1 Mitigates Ischemia and Promotes Tissue Survival and Regeneration

*Aline Zbinden, Shannon L. Layland, Max Urbanczyk, Daniel A. Carvajal Berrio, Julia Marzi, Monika Zauner, Anne Hammerschmidt, Eva M. Brauchle, Katrin Sudrow, Simon Fink, Markus Templin, Simone Liebscher, Gerd Klein, Arjun Deb, Garry P. Duffy, Gay M. Crooks, Johannes A. Eble, Hanna K. A. Mikkola, Ali Nsair, Martina Seifert, and Katja Schenke-Layland**

Supporting Information

Nidogen-1 Mitigates Ischemia and Promotes Tissue Survival and Regeneration

Authors

Aline Zbinden[1], Shannon L. Layland[1], Max Urbanczyk[1], Daniel A. Carvajal Berrio[1,2], Julia Marzi[1,3], Monika Zauner[1], Anne Hammerschmidt[1], Eva M. Brauchle[1,2,3], Katrin Sudrow[4,5], Simon Fink[3], Markus Templin[3], Simone Liebscher[1], Gerd Klein[6], Arjun Deb[7,8,9,10,11], Garry P. Duffy[12], Gay M. Crooks[8,10,13], Johannes A. Eble[14], Hanna K. A. Mikkola[7,8,9,10], Ali Nsair[8,11], Martina Seifert[4,5] and Katja Schenke-Layland[1,2,3,11]*

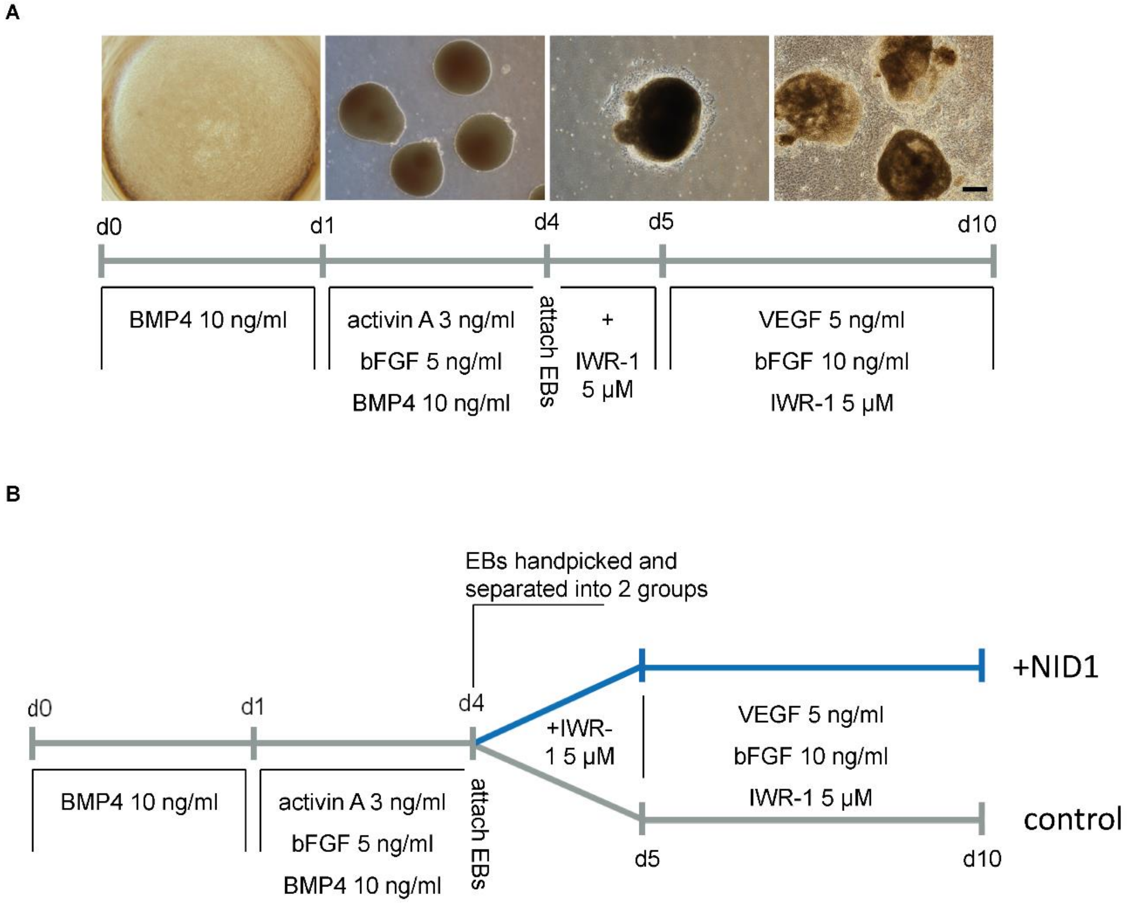

Figure S1. Schematic illustration of the modified cardiovascular differentiation protocol. EBs were derived from hESCs (line H9). **(A)** The differentiation protocol timeline is shown together with the stage-specific additives to the medium. Scale bar equals 100 µm. **(B)** The differentiation protocol using NID1 from day 4 (d4) and control are shown together with the stage-specific additives in the medium.

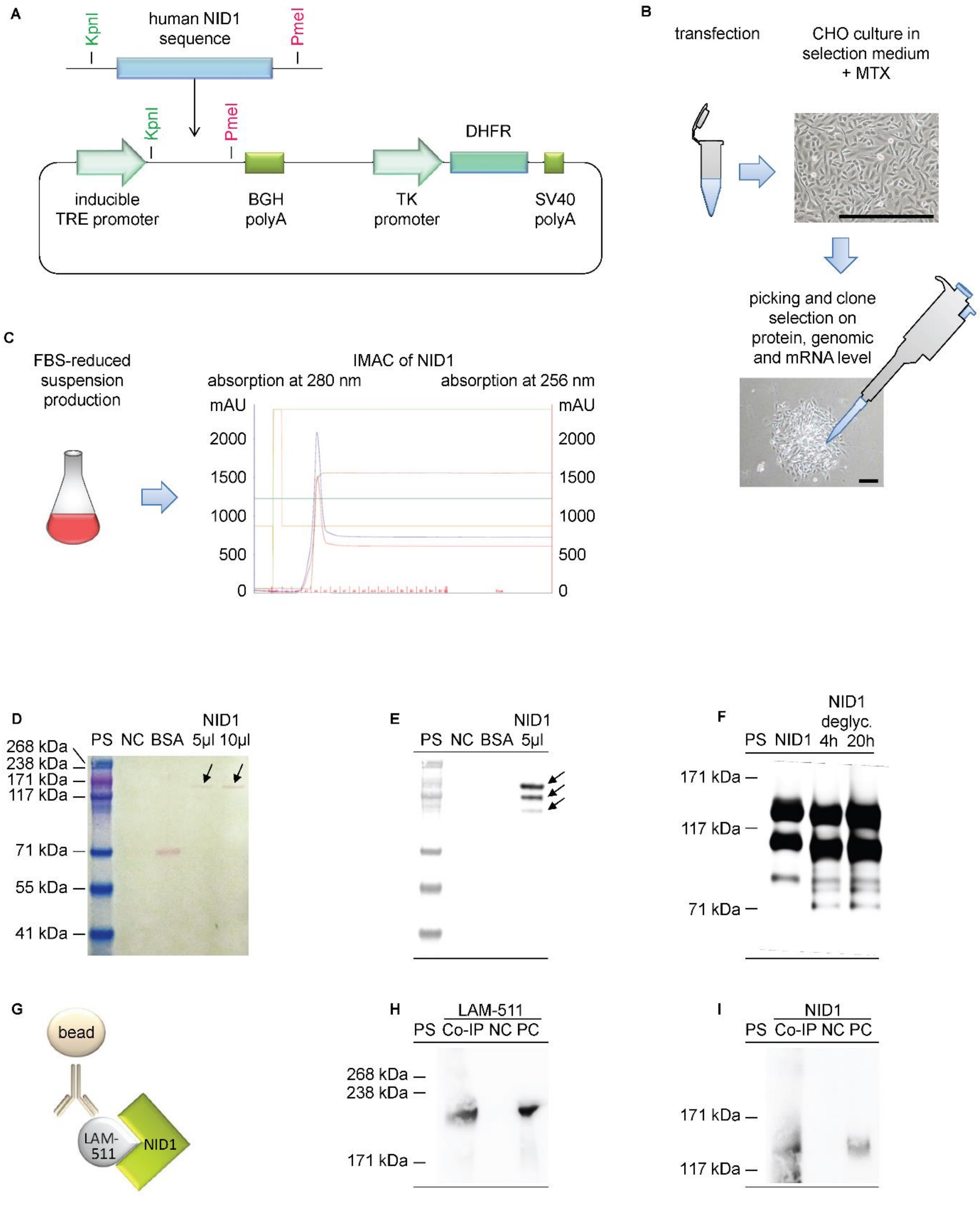

Figure S2. Recombinant NID1 production and characterization. (A) Schematic representation of NID1 production plasmid generation with main components for genomic amplification strategy. **(B)** Schematic depiction of CHO culture transfection with the NID1 production plasmid, MTX selection and clone analysis. Scale bars equal 200 µm. **(C)** Schematic depiction of production clone adaptation to FBS-reduced suspension culture and purification of secreted NID1 using immobilized-metal affinity chromatography (IMAC). Characteristic protein absorption is shown at 280 nm (blue line) and 256 nm (red line) in milli absorption unit (mAU). **(D)** Ponceau-Red staining of a nitrocellulose membrane with IMAC eluates: negative control (NC, 10 µl IMAC eluate of media with 1% FBS, dialyzed), BSA (10 µl IMAC eluate of media with 1% BSA) and 5

or 10 µl IMAC eluate of NID1-containing cell culture media with originally 0.5% FBS, dialyzed. **(E)** Same membrane after discoloring and specific immunodetection of NID1. Arrows indicate 3 NID1 bands at about 140 kDa, 110 kDa and 90 kDa. **(F)** Specific immunodetection of NID1 deglycosylated for 4 hours and for 20 hours shows a shift of the characteristic NID1 bands to lower molecular weight compared to a NID1 control that was not treated with the deglycosylation enzyme, proving the glycosylated state of the recombinant NID1. **(G)** Schematic of Co-IP between interaction partners NID1 and LAM-511. **(H)** Specific immunodetection of the ß1 and ɣ1 chain of LAM-511 in Co-IP, NC (Co-IP w/o LAM antibody) and positive control (PC, LAM-511). **(I)** Specific immunodetection of NID1 in Co-IP, NC (Co-IP w/o LAM antibody) and PC (NID1). The HiMark pre-stained protein standard (PS) was used in all blots.

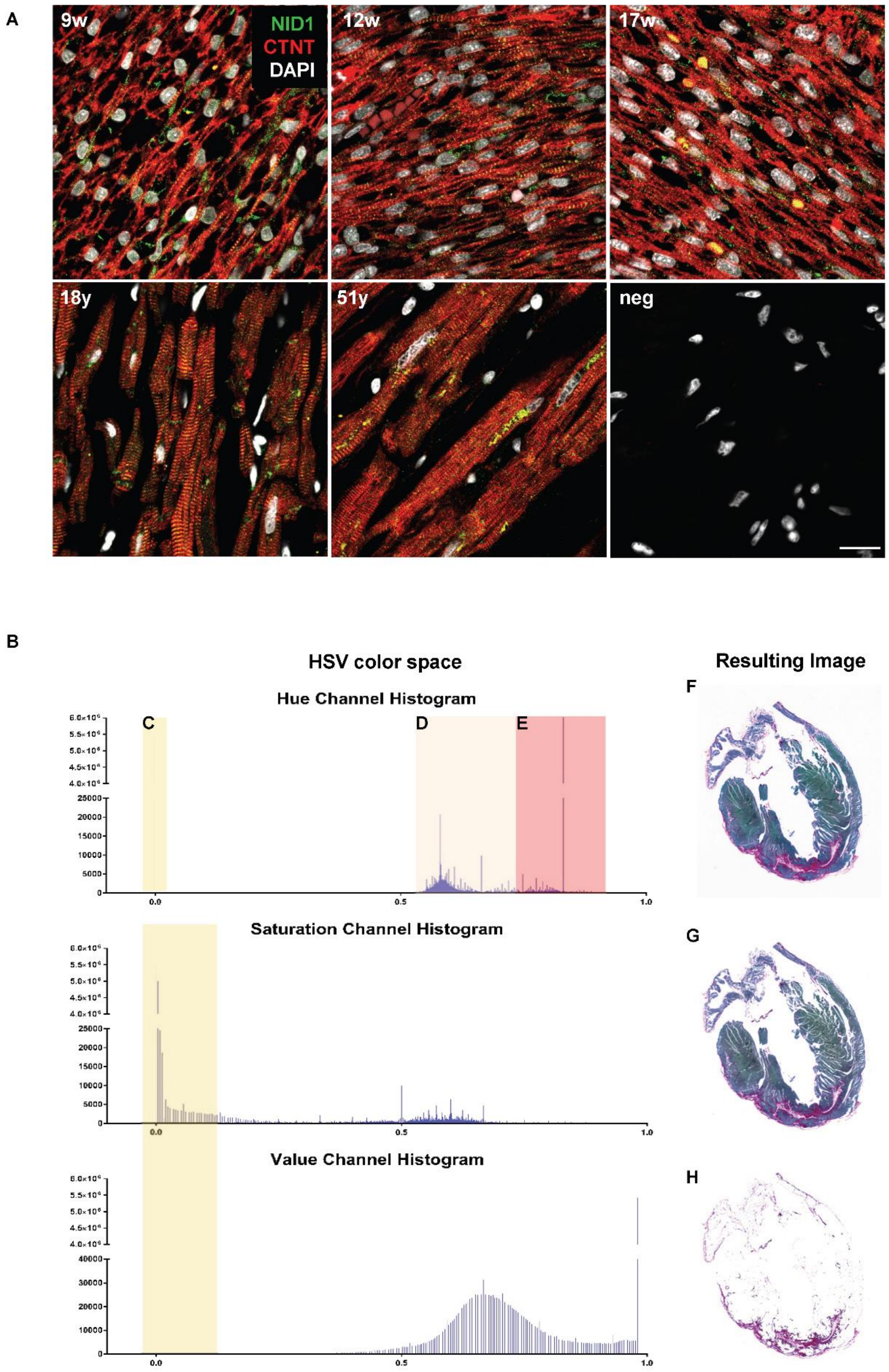

Figure S3. Identification of NID1 as crucial ECM protein in fetal and adult hearts by IF staining & Myocardial scar quantification. (A) NID1 (green) was detected in fetal heart sections 9, 12 and 17 weeks post-fertilization as well as in sections of 18 and 51 year old adult heart tissue. CTNT (red) was used to detect CMs. Cell nuclei were visualized with DAPI (white).

Scale bar equals 20 μm. **(B-H)** HSV color segmentation was performed using a customized MATLAB code. The HSV histogram bands responsible for the background are shown in **(C)**, the Fast Green-positive region labeling the myocardium is shown in **(D)** and, the Picrosirius Red-stained areas corresponding to the scar content are shown in **(E)**. By subtracting band **(C)** from an image of Picrosirius Red and Fast Green **(F)**, an image without background is generated **(G)** and, comparably, the removal of bands **(C)** and **(D)** yields an image of only the scar component **(H)**.

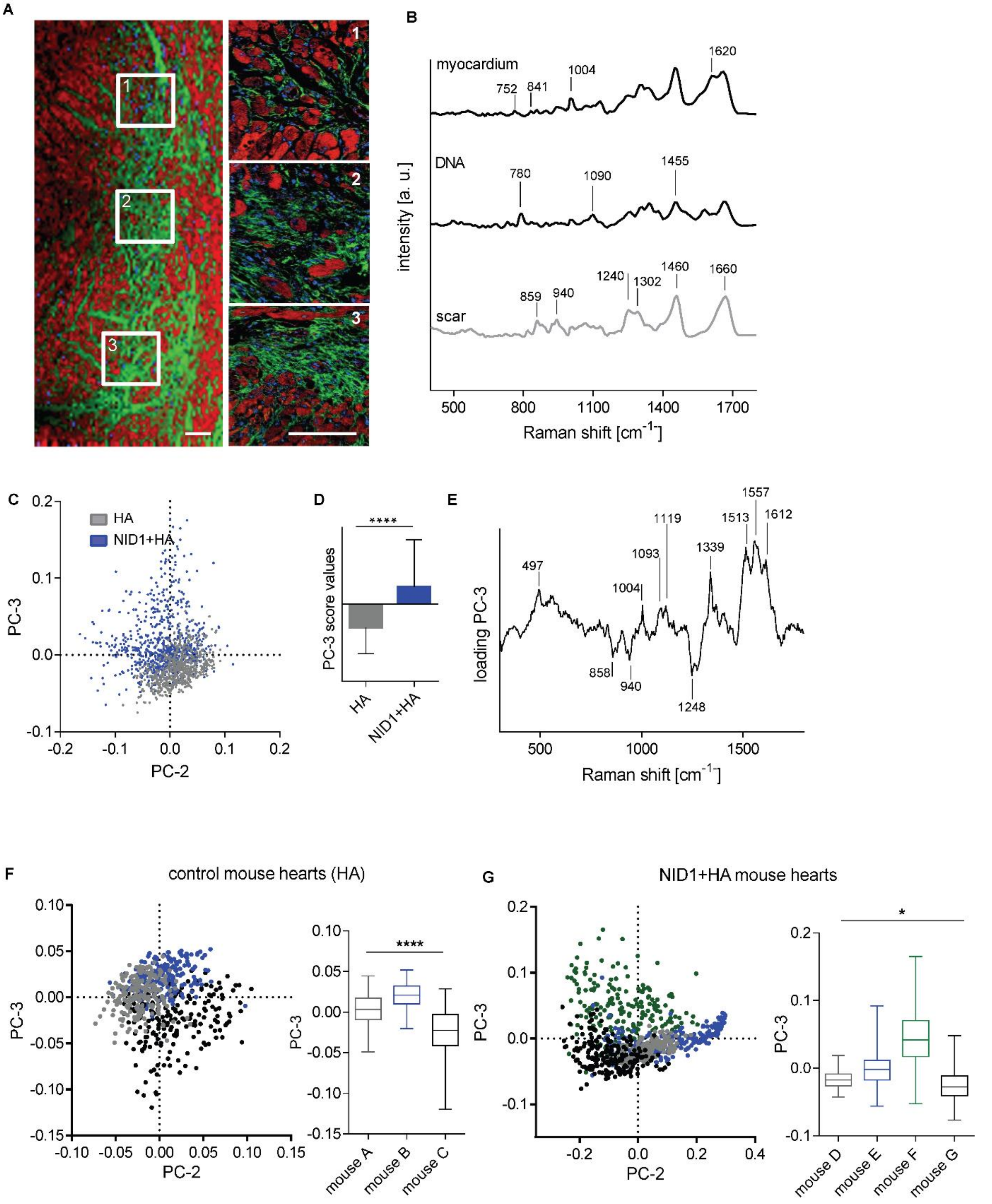

Figure S4. Raman imaging and multivariate analysis of infarcted mouse hearts and single animals. (A) Representative overview of Raman images showing DNA (blue), myocardium (red) and the scar (green). Three regions within the scar area (rectangular boxes: 1-3) were selected for high-resolution Raman images. **(B)** Spectra-associated to the color-coding shown in **(A)**. Based on the signal patterns, the specific ECM composition for the scar area was detected. Scale bar equals 100 µm**. (C,D)** PCA of Raman spectra revealed molecular and structural differences in the scar area of NID1+HA and control hearts. PC score values of spectra from NID1+HA and control

hearts were significantly different, unpaired t-test. **(E)** The loading plot displays typical porphyrin vibrations (1513, 1557, 1612 cm^{-1}) seen in the NID1+HA heart tissue. Collagen peaks (858 and 1248 cm^{-1}) were assigned to the control hearts. PCA of TCA scar component of **(F)** control (HA) and **(G)** NID1+HA mouse hearts. Significant differences between animals are detected in both groups. Kruskal-Wallis test with Dunn's multiple comparisons test. Results are shown as standard box plot diagram. *p<0.05, **p<0.01, ***p<0.001 and ****p<0.0001.

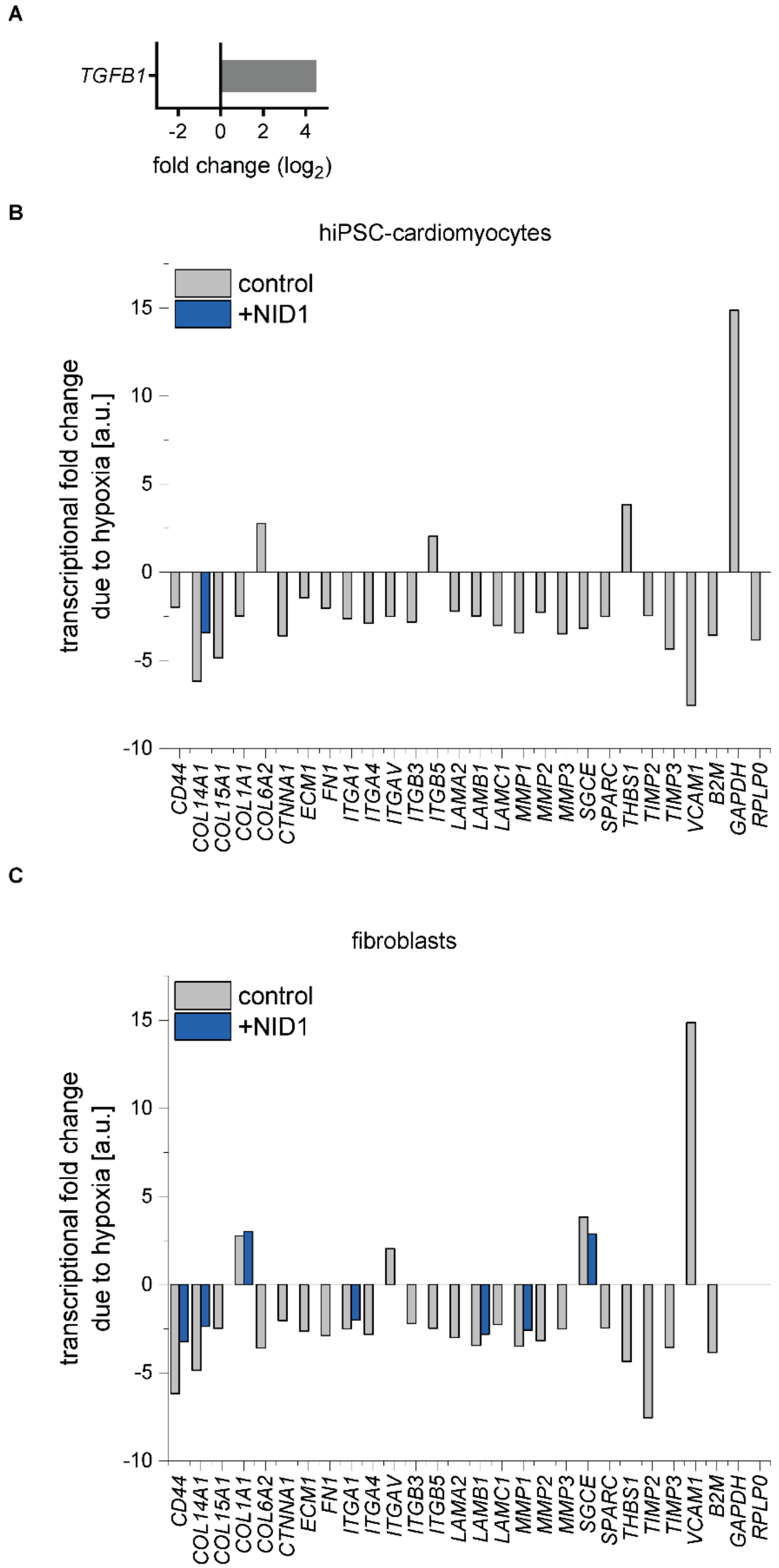

Figure S5. Translational changes in ECM production, degradation and regulation due to hypoxic conditions. (A) Transcriptional changes of *TGFB1* in NID1-treated hiPSC-CMs under normoxic conditions. **(B,C)** Transcriptional changes in gene expression after hypoxic conditions without (control) and with NID1 **(B)** hiPSC-CMs and **(C)** dermal fibroblasts. Gene regulation are shown when fold-regulation > |2|, p-value < 0.05.

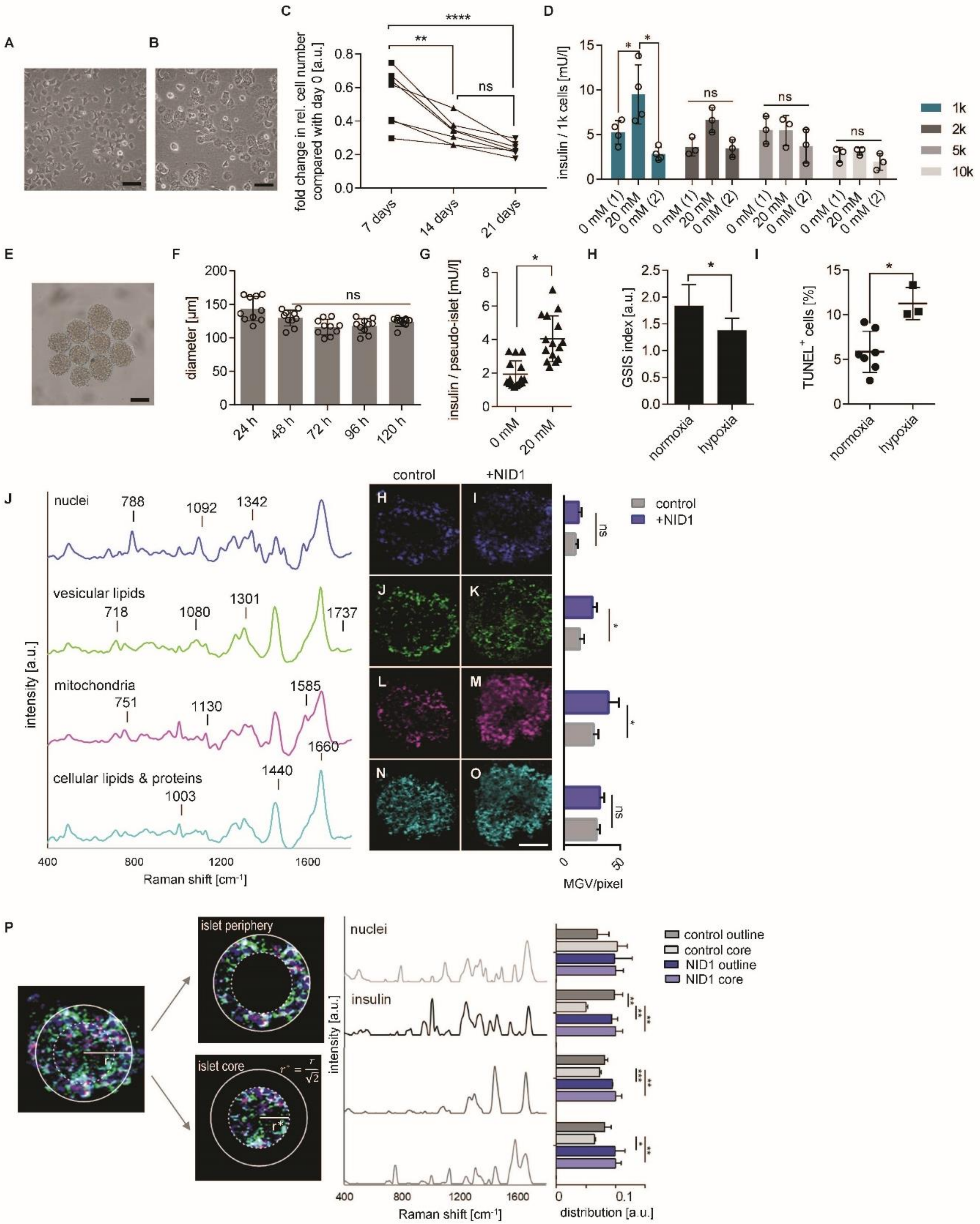

Figure S6. Human pseudo-islet functionality under hypoxic conditions and characterization by in situ Raman imaging and multivariate analysis. (A) Unexcised and **(B)** excised EndoC-βH3 morphology. Scale bars equal 100 µm. **(C)** Reduced cell number over time due to the excision of the proliferation transgene over 21 days. **(D)** GSIS assay (with 0 and 20 mM glucose) of pseudo-islets composed of 1000 (1k), 2000 (2k), 5000 (5k) and 10000 (10k) cells/ pseudo-islet

after 5 days of culture under normoxic conditions. **(E)** 1k pseudo-islet morphology. Scale bar equals 100 µm **(F)** Pseudo-islet size, described as pseudo-islet diameter, is stable from 72h until 5 days under normoxic conditions (n = 10); one-way ANOVA, *p<0.05. **(G)** GSIS assay (with 0 and 20 mM glucose) of pseudo-islets under normoxic conditions at 5 days (n = 15); unpaired t-test, *p<0.05 **(H)** Significant loss of glucose responsiveness under hypoxic conditions, described as the GSIS index (fold increase in insulin release due to the transition from Krebs buffer (0 mM glucose) to 20 mM glucose (n = 10); one-way ANOVA, *p<0.05. **(I)** DNA fragmentation under hypoxic conditions determined by the quantification of $TUNEL^{+}$ cells (n = 3-7); unpaired t-test. **(J)** True component analysis (TCA) identified four major spectral components assigned to nuclei (blue), insulin-transporting lipid vesicles (green), mitochondrial activity (pink) and cellular lipids and proteins (light blue). **(H-O)** False color intensity distribution images for each spectral component in (**H,J,L,N**) PBS controls and (**I,K,M,O**) NID1-treated pseudo-islets. Semi-quantitative analysis by definition of the mean gray value intensity/pixel demonstrated a significant increase in the vesicular lipids and the mitochondrial activity in NID1-treated islets. **(P)** Multivariate curve resolution (MCR) analysis was performed for in-depth analysis of the molecular composition in the periphery and core region of the hypoxic pseudo-islets. Most relevant, components related to nuclei, insulin, lipids and mitochondria were identified and their distribution within both regions were compared in PBS controls and NID1-treated pseudo-islets. The control core region showed a significant decrease in insulin, lipids and mitochondria, whereas the NID1-treated core region showed levels comparable to the peripheral regions (n = 4); *p<0.05, **p<0.01, ***p<0.001.

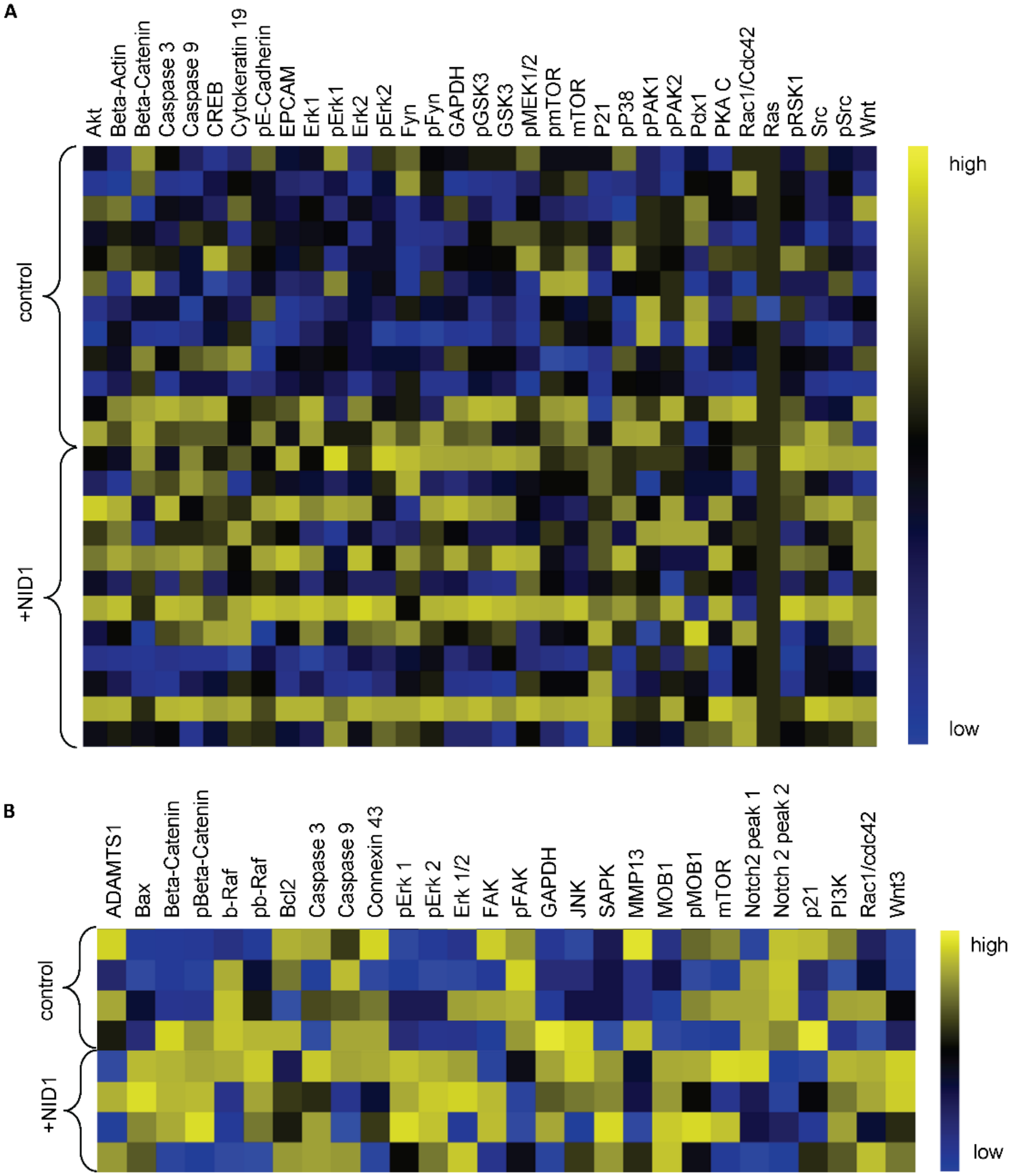

Figure S7. Change in protein expression in NID1-treated cultures. DigiWest®-based protein expression analysis of all tested proteins in (a) NID1-treated pseudo-islets (n = 12) and (b) NID1-treated hiPSC-CMs (n = 4) under hypoxic conditions compared with their respective controls. Data are shown as column-wise and color-coded heatmap from the lowest (blue) to the highest (yellow) expression for each analyte.

	baseline	saline	HA	NID1 + HA
Heart Rate^{min}	605 ± 46	675 ± 18	549 ± 46*	539 ± 45*
LVIDd^{mm}	2.65 ± 0.40	4.77 ± 0.36	3.40 ± 0.44**	2.90 ± 0.18***
LVIDs^{mm}	1.30 ± 0.22	4.29 ± 0.31	2.93 ± 0.51**	2.15 ± 0.18****,#
LVAWd^{mm}	0.73 ± 0.10	0.49 ± 0.01	0.48 ± 0.05	0.51 ± 0.06
LVAWs^{mm}	1.12 ± 0.14	0.52 ± 0.03	0.49 ± 0.04	0.60 ± 0.10
LVPWd^{mm}	0.71 ± 0.03	0.61 ± 0.17	0.53 ± 0.11	0.61 ± 0.07
LVPWs^{mm}	0.98 ± 0.03	0.79 ± 0.17	0.58 ± 0.03	0.70 ± 0.08
LVED^{mm}	2.67 ± 0.31	4.82 ± 0.26	3.52 ± 0.42**	2.82 ± 0.21****
LVES^{mm}	1.27 ± 0.20	4.26 ± 0.30	3.04 ± 0.43**	2.22 ± 0.18****,#
EDV$^{\mu L}$	26.89 ± 7.50	108.80 ± 13.57	52.39 ± 15.62***	30.30 ± 5.69****
ESV$^{\mu L}$	4.18 ± 1.49	81.83 ± 13.34	36.91 ± 13.01***	16.86 ± 3.57****
EF$^{\%}$	84.88 ± 3.08	24.96 ± 4.75	30.17 ± 4.31	44.63 ± 1.57***,##
FS$^{\%}$	52.56 ± 3.70	11.54 ± 2.34	13.80 ± 2.10	21.12 ± 0.78**,#

Table S1. Echocardiography analysis 28 days after MI/R. Baseline is the pre-MI/R measurement of the NID1+HA mice. Data are presented as means ± standard error of the mean (SEM). *p<0.05, **p<0.01, ***p<0.001, ****p<0.0001 saline vs. HA and NID1+HA, #p<0.05, ##p<0.01 HA vs. NID1+HA; individual one-way ANOVA with Tukey's multiple comparison test, baseline (n = 4), saline (n = 3), HA (n = 3), NID1+HA (n = 4). LVIDd: left ventricular internal dimension at end diastole, LVIDs: left ventricular internal dimension at end-systole, LVAWd: left ventricular anterior wall thickness in diastole, LVAWs: left ventricular anterior wall thickness in systole, LVPWd: left ventricular posterior wall dimensions in diastole, LVPWs: left ventricular posterior wall dimensions in systole, LVED: left ventricle end-diastolic diameter, LVES: left ventricle end-systolic diameter, EDV: end-diastolic volume, ESV: end-systolic volume, EF: ejection fraction, FS: fractional shortening.

Raman shift [cm^{-1}]	Assignment
497	glycogen
718	C-N / nucleotide peak
751	mitochondrial DNA
752	porphyrin
780	DNA
788	DNA
841	Polysaccharides
858-9	proline, hydroxyproline
940	C-C backbone
1003	phenylalanine
1004	phenylalanine
1080	typical phospholipids
1090-3	phosphodioxy / DNA
1119	C-C stretch in lipids
1130	phospholipid structural changes / Mitochondrial activity
1240	lipids
1247-8	collagens, amide III
1301	lipids, fatty acids, triglycerides
1302	collagens
1339	tryptophan, CH2, CH3 wagging - bending in lipids/proteins
1342	guanine (DNA/RNA)
1440	CH2, CH3 deformation (lipids)
1455	deoxyribose
1460	CH2, CH3 deformation of lipids and collagen
1513	cytosine, porphyrin
1557	porphyrin v(C=C)
1585	mitochondria /mitochondrial activity
1612	C=C stretching (ring)
1620	porphyrin v(C=C)
1660	amide I
1737-8	lipids

Table S2. Raman-associated peaks. Raman peaks in cm^{-1} and their assigned molecular structure.

antibodies for DigiWest	species	order information
Beta-Actin	rabbit IgG	A1978, Sigma
ADAMTS1	rabbit IgG	12897, Cell Signaling
Akt	rabbit IgG	4685, Cell Signaling
Bax	rabbit IgG	2772, Cell Signaling
Bcl2	rabbit IgG	2872, Cell Signaling
Beta-Catenin	rabbit IgG	8480, Cell Signaling
Beta-Catenin	rabbit IgG	8814, Cell Signaling
Beta-Catenin - phospho Ser552	rabbit IgG	9566, Cell Signaling
b-Raf	rabbit IgG	07-583, Upstate
b-Raf - phospho Ser445	rabbit IgG	2696, Cell Signaling
Caspase 3	rabbit IgG	9662, Cell Signaling
Caspase 9	mouse IgG1	9508, Cell Signaling
Connexin 43	rabbit IgG	3512, Cell Signaling
CREB - phospho Ser133	rabbit IgG	9198, Cell Signaling
EpCAM	rabbit IgG	3599, Cell Signaling
Erk1/2 (MAPK p44/42)	rabbit IgG	4695, Cell Signaling
Erk1/2 (MAPK p44/42) - phospho Thr202/Tyr204	rabbit IgG	4370, Cell Signaling
E-cadherin	rat IgG	sc-59778, Santa Cruz
E-cadherin – phospho Ser838/Ser840	rabbit IgG	2239-1, Epitomics
FAK1	rabbit IgG	3285, Cell Signaling
FAK1 - phospho Tyr397	rabbit IgG	8556, Cell Signaling
Fyn	rabbit IgG	4023, Cell Signaling
Fyn – phospho Tyr530	rabbit IgG	orb99261, biorbyt
GAPDH	rabbit IgG	5174, Cell Signaling
GSK3 alpha/beta - phospho Ser21/Ser9	rabbit IgG	9331, Cell Signaling
GSK3 beta	rabbit IgG	9315, Cell Signaling
GSK3 beta - phospho Ser9	rabbit IgG	9336, Cell Signaling
JNK/SAPK	rabbit IgG	9252, Cell Signaling
MEK1/2 - phospho Ser217/Ser221	rabbit IgG	9154, Cell Signaling
MMP13	mouse IgG1	MAB511, R&D Systems
MOB1	rabbit IgG	3863, Cell Signaling
MOB1 - phospho Thr35	rabbit IgG	8699, Cell Signaling
mTOR (FRAP)	rabbit IgG	2983, Cell Signaling
mTOR (FRAP)- phospho Ser2448	rabbit IgG	2971, Cell Signaling
Notch 2	rabbit IgG	5732, Cell Signaling
P21	rabbit IgG	2947, Cell Signaling
P38 MAPK - phospho Thr180/Tyr182	rabbit IgG	4511, Cell Signaling
PAK 1/2 - phospho Ser144/Ser141	rabbit IgG	2606, Cell Signaling
Pdx1	rabbit IgG	5679, Cell Signaling
PI3-kinase p85 alpha	rabbit IgG	ab40755, abcam
PKA C alpha	rabbit IgG	4782, Cell Signaling
Rac1/Cdc42	rabbit IgG	4651, Cell Signaling
Ras	rabbit IgG	8955, Cell Signaling
RSK 1 (p90RSK) - phospho Thr573	rabbit IgG	ab62324, abcam
Src	rabbit IgG	2108, Cell Signaling
Src – phospho Tyr527	rabbit IgG	2105, Cell Signaling
Wnt3A	rabbit IgG	09-162, Millipore
**antibodies for DigiWest**	**species**	**order information**
dk-α-rb IgG (H+L) - RPE		#711-116-152, Jackson
dk-α-ms IgG (H+L) - RPE		#715-116-151, Jackson
gt-α-rt IgG (H+L) - RPE		#112-116-143, Jackson
Streptavidin - RPE		#016-110-084, Jackson

antibodies for IF staining	species	dilution	order information
NID1	rabbit IgG	1:100	Sc-33141, Santa Cruz
NID1	rabbit IgG	1:1000	kind gift from F. Zaucke
NID2	rabbit IgG	1:1000	kind gift from F. Zaucke
FN	rabbit IgG	1:500	A0245, Dako
POSTN	rabbit IgG	1:100	HPA012306, Sigma-Aldrich
LAM	rabbit IgG	1:50	ab11575, Abcam
COL4	rabbit IgG	1:250	ab6586, Abcam
COL1	rabbit IgG	1:75	R1038, Acris
αSMA-FITC	mouse IgG2a	1:500	F3777, Sigma-Aldrich
CD31	rat IgG2a	1:20	DIA-310, Dianova
MF20	mouse IgG2b	1:50	MF20, Developmental Studies Hybridoma Bank
CTNT	rabbit IgG	1:3000	HPA015774, Sigma-Aldrich
CTNT	mouse IgG1	1:600	NB200-412, Novus Biologicals
TUJ1	rabbit IgG	1:2000	802001, BioLegend
NESTIN	rabbit IgG	1:200	839801, BioLegend
MAP2	rabbit IgG	1:500	ab5622, Merck-Millipore
INS	guinea-pig IgG	1:200	A0564, Dako
E-cadherin	mouse IgG2a	1:250	ab76055, Abcam
CASP-3	rabbit IgG	1:100	ab13847, Abcam
rabbit IgG-AF488	goat	1:250	A-11034, Invitrogen
rat IgG2a-AF647	goat	1:250	A-21247, Invitrogen
mouse IgG1-AF594	goat	1:250	A-21125, Invitrogen
mouse IgG2a-AF488	goat	1:250	A-21131, Invitrogen
mouse IgG2b-AF594	goat	1:250	A-21145, Invitrogen
guinea-pig IgG-AF594	goat	1:250	A-11076, Invitrogen
**antibodies for WB**	**species**	**dilution**	**order information**
NID1	rabbit IgG	1:400	sc-33141, Santa Cruz
LAM	Rabbit IgG	1:500	ab11575, Abcam
anti-rabbit IgG-HRP	goat	1:4000	ab6721, Abcam

Primer		Sequence
Human NID1 5'	QhNID1-389F<br>QhNID1-495R	CCGCTGAGTGCGTGCACAGA<br>GTCCCGAGAAGGGCCCTGGT
Human NID1 3'	QhNID1-2015F<br>QhNID1-2160R	CCACGGCTGCGATACCAACGCCGCC<br>CAGGATGTGCTCTCTCTCGTGCTGG
Hamster B-Actin	QhamB-Actin-F<br>QhamB-Actin-R	GGCGCTTTTGACTCAGGACTTTA<br>GGGATGTTTGCTCCAACCGA
Hamster p53	Qham-p53-F<br>Qham-p53-R	CATGCCGAATACCTGGATGACAAG<br>GCAAATCAAACCCTGTCTTCAACC
human αSMA	Hs_ACTA2_1_SG	Qiagen, QT00088102
human GAPDH	Hs_GAPDH_2_SG	Qiagen, QT01192646
RT² Profiler PCR Arrays	Extracellular Matrix & Adhesion Molecules	Qiagen, PAHS-013Z

Table S3. List of antibodies and primer sequences.
